# Genome-wide identification and bioinformatics analysis of the WRKY transcription factors and screening of candidate genes for anthocyanin biosynthesis in azalea (*Rhododendron simsii*)

**DOI:** 10.3389/fgene.2023.1172321

**Published:** 2023-05-10

**Authors:** Cheng Wang, Dan Ye, Yan Li, Peiling Hu, Run Xu, Xiaojing Wang

**Affiliations:** ^1^ Key Laboratory for Quality Control of Characteristic Fruits and Vegetables of Hubei Province, College of Life Science and Technology, Hubei Engineering University, Xiaogan, China; ^2^ Department of Biology and Chemical Engineering, Weihai Vocational College, Weihai, China; ^3^ Key Laboratory of Plant Resource Conservation and Germplasm Innovation in Mountainous Region (Ministry of Education), Guizhou University, Guiyang, China

**Keywords:** azalea, WRKY, phylogenetic analysis, anthocyanin biosynthesis, expression pattern

## Abstract

WRKY transcription factors have been demonstrated to influence the anthocyanin biosynthesis in many plant species. However, there is limited knowledge about the structure and function of *WRKY* genes in the major ornamental plant azalea (*Rhododendron simsii*). In this study, we identified 57 *RsWRKY* genes in the *R. simsii* genome and classified them into three main groups and several subgroups based on their structural and phylogenetic characteristics. Comparative genomic analysis suggested *WRKY* gene family has significantly expanded during plant evolution from lower to higher species. Gene duplication analysis indicated that the expansion of the *RsWRKY* gene family was primarily due to whole-genome duplication (WGD). Additionally, selective pressure analysis (*Ka*/*Ks*) suggested that all *RsWRKY* duplication gene pairs underwent purifying selection. Synteny analysis indicated that 63 and 24 pairs of *RsWRKY* genes were orthologous to *Arabidopsis thaliana* and *Oryza sativa*, respectively. Furthermore, RNA-seq data was used to investigate the expression patterns of *RsWRKYs*, revealing that 17 and 9 candidate genes may be associated with anthocyanin synthesis at the bud and full bloom stages, respectively. These findings provide valuable insights into the molecular mechanisms underlying anthocyanin biosynthesis in *Rhododendron* species and lay the foundation for future functional studies of *WRKY* genes.

## 1 Introduction

The *Rhododendron* genus (Ericaceae), which has more than 1,000 species and 30,000 cultivars, is mostly found in the Northern Hemisphere and is renowned for the exceptional beauty and variety of its corollas (Yang F. S. et al., 2020; Olech et al., 2020). *Rhododendron* species such as *Rhododendron simsii* and *Rhododendron delavayi* Franch are globally famous as ornamental plants. Flower color is the primary consideration in the breeding and creation of new ornamental varieties. The majority of earlier studies on pigment analysis for azalea flowers concentrated on the pigment type and composition percentage (Yang F. S. et al., 2020). Their underlying genetic and regulatory mechanisms, however, are little understood.

Anthocyanins, a subclass of flavonoids, are crucial secondary metabolites in plants and are regarded as one of the primary pigments in floral color development, causing its red, pink, blue, purple and dark color (Yoshida et al., 2009; Xu et al., 2015). The biosynthesis of anthocyanins is mediated by two categories of genes. The first category consists of structural genes that code for the enzymes that catalyze each stage of the biosynthesis process, including phenylalanine-ammonia lyase (PAL), anthocyanidin synthase (ANS), UDP-glucose:flavonoid-3-O-glucosyltransferase (UFGT), dihydroflavonol-4-reductase (DFR), chalcone synthase (CHS), rhamnosyl transferase (RT), chalcone isomerase (CHI), 4-coumaryl-CoA ligase (4CL), flavonoid 3′-hydroxylase (F3′H) and flavanone 3-hydroxylase (F3H) (Forkmann and Martens, 2001; Debeaujon et al., 2003; Tanaka et al., 2009; Chen et al., 2012; Zhang B. et al., 2021). The majority of the structural genes associated with the biosynthesis of anthocyanins have been thoroughly studied and characterized in numerous plants. The second category consists of regulatory genes that encode different transcription factors (TFs), including WD40, bHLH, and MYB, which could control the transcription of structural genes (Zhao and Tao, 2015; Yan et al., 2021). These TFs could combine to form the ternary MYB-bHLH-WD40 (MBW) complex, which is widely recognized as the primary controller of anthocyanin deposition in many plants (Ramsay and Glover, 2005; Li, 2014; Cui et al., 2021). Recently, it was discovered that additional TFs, like WRKY, contribute to the synthesis of anthocyanins (Yang et al., 2015; Lloyd et al., 2017; An et al., 2019; Li C. et al., 2020; Cong et al., 2021).

The WRKY TF family is one of the largest transcription factor families in higher plants. According to earlier research, the WRKY family is essential for controlling plant development, growth, and responses to both biotic and abiotic stresses (Rushton et al., 2010; Rinerson et al., 2015). Recent investigations further demonstrated the role of WRKY TFs in the regulation of anthocyanin biosynthesis. For example, AtWRKY41 and its ortholog BnWRKY41-1 suppress the production of anthocyanins in *Arabidopsis thaliana* (Duan et al., 2018)*.* In red pear (*Pyrus* L.), PpWRKY44 could directly activate the PpMYB10 promoter to positively control light-induced anthocyanin biosynthesis (Alabd et al., 2022). PbWRKY75 might enhance the expression of *PbMYB10b* and the genes responsible for the late biosynthesis of anthocyanins (*PbDFR* and *PbUFGT*), increasing the biosynthesis of anthocyanin in pears (Cong et al., 2021). Moreover, PyWRKY26 and PybHLH3 co-targeted the PyMYB114 promoter to control anthocyanin production and transport (Li C. et al., 2020). In apple (*Malus domestica* L.), the accumulation of anthocyanins could be greatly aided by MdWRKY11, and the expression of several MYB TFs and structural genes also rose dramatically (Liu W. et al., 2019). The TF cascade MdHY5-MdWRKY41-MdMYB controls the synthesis of proanthocyanins and anthocyanins (Mao et al., 2021). When exposed to ultraviolet light, MdWRKY72 may enhance the synthesis of anthocyanins in transgenic apple calli (Hu et al., 2020). Additionally, in collaboration with MdMYB1, MdWRKY40 was discovered to facilitate wounding-induced anthocyanin synthesis. (An et al., 2019). Overexpressing of *McWRKY71* increased the resistance of Malus crabapple to ozone stress and promoted the accumulation of anthocyanins and proanthocyanidins (Zhang et al., 2022). In eggplant (*Solanum melongena* L.), SmWRKY44 and SmMYB1 can interact to enhance the production of anthocyanins (He et al., 2021). In potato (*Solanum tuberosum* L.) tubers, StWRKY13 promotes the production of anthocyanins (Zhang H. et al., 2021). These findings show that WRKY TFs are important for anthocyanin synthesis and accumulation.

WRKY TFs are characterized by the presence of one or two WRKY domains. The WRKY domain is around 60 amino acids long and contains the highly conserved sequence “WRKYGQK” and a zinc-finger motif (Eulgem et al., 2000). The heptapeptide motif ‘WRKYGQK’ and the zinc finger motif are both necessary for the WRKY protein to bind to the consensus cis-acting element W-box (C/TTGACC/T) (Zhang and Wang, 2005). WRKY TFs can be separated into 3 main groups and 7 subgroups according to the quantity of WRKY domains and zinc-finger motif characteristics. Typically, Group I contain two WRKY domains and one C2H2 motif (C–X4-5–C–X22-23–H–X–H, where “X” is an amino acid). Group II were found to possess a single WRKY domain and a C2H2 motif, this group has been further divided into five subgroups (IIa–IIe): IIa (CX5CPVKKK(L/V) Q), IIb (CX5CPVRKQVQ), IIc (CX4C), IId (CX5CPARKHVE) and IIe (CX5CPARK (Q/M) V (E/D)). Group III possesses a WRKY domain and a C2HC motif (C–X7–C–X23–H–X–C) (Eulgem et al., 2000; Rushton et al., 2010). Numerous plants, including *Arabidopsis* (Eulgem et al., 2000), wheat (*Triticum aestivum* L.) (Ning et al., 2017), tomato (*Solanum lycopersicum* L.) (Huang et al., 2012), cotton (*Gossypium raimondii* and *Gossypium hirsutum*) (Dou et al., 2014), poplar (*Populus trichocarpa* L.) (He et al., 2012), pineapple (*Ananas comosus* L. Merrill) (Xie et al., 2018), soybean (*Glycine max* L. Merrill) (Yu et al., 2016), peanut (*Arachis hypogaea* L.) (Song et al., 2016), and maize (*Zea mays* L.) (Hu et al., 2021) have had the full genome level identification of their WRKY TFs.

There is, however, limited knowledge, particularly on the *WRKY* genes of the azalea’s role in the synthesis of anthocyanins. Clarifying the evolution and potential roles of *WRKY* genes in azalea requires more research. Recently, the generation of azalea genome has presented an exceptional opportunity to identify the WRKY TF family across the entire genome (Yang F. S. et al., 2020). In this study a genome-wide analysis of azalea *WRKY* genes was accomplished, 57 *WRKY* genes in all were identified. Subsequently, we examined the categorization, phylogenetic relationships and gene duplications of the azalea *WRKY* (*RsWRKY*) genes. Moreover, the candidate *RsWRKYs* that linked to anthocyanin biosynthesis were identified according to RNA-seq data in three *Rhododendron* species with various flower colors. The findings of this research improve our knowledge of the *RsWRKY* gene family and will facilitate the genetic improvement of flower color in azalea.

## 2 Materials and methods

### 2.1 Investigation of *WRKY* gene family in azalea and other plant species

The whole protein sequence data of azalea were retrieved from RPGD (Rhododendron Plant Genome Database, http://bioinfor.kib.ac.cn/RPGD/) (Yang F. S. et al., 2020). The WRKY HMM profile (PF03106) was used as a query to search predicted RsWRKY proteins using the HMMER 3.0 software (Eddy, 2011). Subsequently, putative RsWRKY proteins were submitted to SMART (Letunic et al., 2021) and Pfam (Mistry et al., 2021) databases to confirm the presence of typical WRKY domains, only sequences with at least one WRKY characteristic amino acid sequence can be retained.

The WRKY proteins of following species were retrieved from prior studies: *Arabidopsis thaliana* (Eulgem et al., 2000), *Brassica napus* (He et al., 2016), *Malus domestica* (Qin et al., 2022), *Camellia sinensis* (Wang et al., 2019), *Cucumis sativus* (Chen et al., 2020b), *Physcomitrella patens* (Rensing et al., 2008), *Theobroma cacao* (Silva Monteiro de Almeida et al., 2017), *Medicago truncatula* (Kumar et al., 2016), *Cyanidioschyzon merolae* (Dou et al., 2014), rice (*Oryza sativa*) (Ramamoorthy et al., 2008), *Ginkgo biloba* (Cheng et al., 2019), *Chlamydomonas reinhardtii* (Rinerson et al., 2015), *Vitis vinifera* (Guo et al., 2014), *Solanum melongena* (Yang Y. et al., 2020), *Azolla filiculoides* (Zhao et al., 2021), *Salvinia cucullate* (Zhao et al., 2021), *Selaginella moellendorffii* (Rinerson et al., 2015), *Moringa oleifera* (Zhang et al., 2019), *Solanum lycopersicum* (Huang et al., 2012), *Sorghum bicolor* (Baillo et al., 2020) and *Populus trichocarpa* (Jiang et al., 2014). The evolutionary relationships of these species were determined according to the PGDD database (Lee et al., 2013) and previous study (Liu et al., 2021).

### 2.2 Classification of the RsWRKY TFs and phylogenetic analysis

According to the quantity of WRKY domains and zinc-finger motif features, WRKY TFs can be divided into 3 main groups and 7 subgroups (Eulgem et al., 2000; Rushton et al., 2010). Group I contain two WRKY domains and one C2H2 motif (C–X4-5–C–X22-23–H–X–H). Group II were found to possess a single WRKY domain and a C2H2 motif, this group has been further divided into five subgroups (IIa–IIe): IIa members contain CX5CPVKKK (L/V) Q structure, IIb members contain CX5CPVRKQVQ structure, IIc members contain CX4C structure, IId members contain CX5CPARKHVE structure, and IIe members contain CX5CPARK (Q/M) V (E/D) structure. Group III possesses a WRKY domain and a C2HC motif (C–X7–C–X23–H–X–C).

A phylogenetic tree was built using the following five species: Azalea, *Rhododendron ovatum* (a related asteroid eudicot), *A. thaliana* (a rosid eudicot), *Aquigelia coerulea* (a basal eudicot), and *Amborella trichopoda* (a basal angiosperm). The WRKY proteins from *R. ovatum*, *A. coerulea* and *A. trichopoda* were obtained using the same method as described in Section 2.1, whereas the AtWRKY proteins from *A. thaliana* were collected from prior investigations (Eulgem et al., 2000). The WRKY protein sequences from five species were aligned using T-Coffee (Notredame et al., 2000). The alignment sequences were then edited with the Jalview program (Waterhouse et al., 2009), with all non-WRKY domain sequences removed. A phylogenetic tree was constructed based on the WRKY domain sequences using IQ-TREE (Minh et al., 2020) with a maximum-likelihood approach and a bootstrap value of 5,000. Finally, iTOL (Letunic and Bork, 2021) was used to visualize and embellish the phylogenetic tree.

### 2.3 Gene duplication analysis

DupGen_finder (Qiao et al., 2019) was utilized to distinguish between whole-genome duplication (WGD), tandem duplication, transposed duplication, proximal duplication and dispersed duplication with the default settings. Duplicated *RsWRKY* gene pairs were visualized using TBtools software (Chen et al., 2020a). Using the KaKs_Calculator (version 2.0) (Wang et al., 2010), *Ka*, *Ks* and *Ka*/*Ks* values were calculated for all duplication gene pairs. The synteny of the *WRKY* genes between Azalea and other plants was examined using MCScanX (Wang et al., 2012), and the findings were presented visually using TBtools software (Chen et al., 2020a).

### 2.4 Expression analysis of *RsWRKY* genes in three different colors of azaleas by RNA-seq

The expression patterns of the *RsWRKY* genes were investigated using RNA-seq data obtained from azalea flower samples of three different colors and at two different stages of floral development. The RNA-seq data have been deposited in the SRA database (http://www.ncbi.nlm.nih.gov/sra) under the identifier PRJNA952839. The three azalea varieties are *Rhododendron wardii* var. puralbum (possessing white color flower, WF), *Rhododendron hybridum* Ker Gawl (possessing red color flower, RF), and *Rhododendron simsii* Planch (possessing pink color flower, PF). The flower samples for RNA-seq were collected at the bud and full bloom stages from plants grown in a controlled-environment growth chamber with a temperature of 25/18°C (day/night), 16/8 light/dark cycle, and 60%–70% relative humidity. Fragments per kilobase of exon model per million mapped reads (FPKM) was used to measure the transcript abundance of *RsWRKY* genes. The gene expression data for each *RsWRKY* were normalized by log2 (FPKM) and shown as heat maps using the TBtools software (Chen et al., 2020a). Genes that showed absolute |fold change| ≥ 2 (log2 |fold change| ≥ 1) for differential expression between two azalea varieties were defined as differentially expressed genes (DEGs).

The following comparative strategy was used to identify candidate genes associated with flower coloration. First, the comparison between colored and white flowers (WF versus RF, WF versus PF) was performed using gene expression data from the same stages of floral development to generate two DEG sets (both up- and downregulated DEGs were included). The two DEG sets were then intersected to produce the final DEGs. The candidate genes at the bud stage and full bloom stage were derived using this comparative strategy, respectively.

### 2.5 Confirmation of RNA-seq analysis in the flowers of three azalea varieties

The expression levels of 9 candidate *RsWRKY* genes at the full bloom stage were assessed by qRT-PCR to confirm the validity of RNA-seq results. The RNA used in the RT-PCR experiments was the same as the RNA used in the RNA-seq analysis mentioned above. The qRT-PCR Primers were designed by Beacon Designer 8 (Supplementary Table S1). According to our RNA-seq data and a previous study (De Keyser et al., 2013), the expression levels of *RsGAPDH* (Rhsim12G0106200) and *RsEF1α* (Rhsim02G0008200) were stable in different varieties with different flower colors and at different developmental stages, therefore these two genes were used as internal reference genes. The StepOnePlus™ System (Applied Biosystems, Foster City, CA, United States) was used to perform the qRT-PCR with the following cycling profile: 95°C for 30°s, 95°C for 5 s (40 cycles), 60°C for 30 s. Each qRT-PCR test employed three biological and three technical replicates. The 2^−ΔΔCt^ method was used to calculate the outcomes (Livak and Schmittgen, 2001). The qRT-PCR reaction mixture was performed with a TB Green^®^ Premix Ex Taq™ kit (TaKaRa, Dalian, China).

### 2.6 GO enrichment analysis of candidate *RsWRKY* genes

GOATOOLS (http://github.com/tanghaibao/GOatools) (Klopfenstein et al., 2018) was utilized to annotate candidate *RsWRKY* genes and Fisher’s exact test was employed for biological function enrichment analysis. To minimize false positives, the Bonferroni multiple testing correction was applied, with significance defined as a corrected *p*-value (Padjust) of less than 0.05. The results were presented using a bubble dot diagram, which was generated using the website (www.bioinformatics.com.cn).

### 2.7 Three-dimensional protein structure prediction

Three-dimensional (3D) structures of candidate RsWRKY proteins were modeled on the basis of homology modeling using SWISS-Model (https://swissmodel.expasy.org/) (Waterhouse et al., 2018). In addition, the model quality was evaluated by global model quality estimation (GMQE) (Waterhouse et al., 2018) and QMEANDisCo global (Studer et al., 2020). The GMQE and QMEANDisCo global score were between 0 and 1, and the higher score indicates the model is more reliable.

## 3 Result

### 3.1 Identifcation of the *RsWRKYs*

We totally identified 57 *RsWRKY* gene members, which were further renamed as *RsWRKY1* to *RsWRKY57* according to their chromosomal positions on Chr1-13 and the order of scaffolds (Table 1). The identification outcomes showed one gene that was different from the study by Wan et al. (2022). Our findings contain the RhsimUnG0199600 gene, while their findings include the Rhsim10G0148600 gene. Due to the presence of a complete WRKY domain, the RhsimUnG0199600 gene is undoubtedly a *WRKY* gene. However, the WRKY domain in the Rhsim10G0148600 gene had almost entirely been gone, therefore we removed it throughout the screening process.

**TABLE 1:** List of the identified *RsWRKY* genes and their related information.

Name	Gene id	Group	ORF (aa)	Conserved heptapeptide	Zinc-finger type	Domain number	Gene type	Ortholog in *A. thaliana*
RsWRKY1	Rhsim01G0033600	I	397	WRKYGQK	C2H2	2	Dispersed	AT4G30935
				WRKYGQK				
RsWRKY2	Rhsim01G0241100	I	548	**WRKYGEK**	C2H2	2	WGD	AT2G38470
				WRKYGQK				
RsWRKY3	Rhsim01G0268400	III	346	WRKYGQK	Lost	1	Transposed	AT5G28650
RsWRKY4	Rhsim02G0002700	IIc	213	WRKYGQK	C2H2	1	WGD	At1G64000
RsWRKY5	Rhsim02G0146200	IIe	293	WRKYGQK	C2H2	1	Transposed	At2G34830
RsWRKY6	Rhsim02G0213700	IIa	316	WRKYGQK	C2H2	1	Tandem	At4G31800
RsWRKY7	Rhsim02G0213800	IIa	291	WRKYGQK	C2H2	1	Tandem	At2G25000
RsWRKY8	Rhsim03G0153300	III	315	WRKYGQK	C2HC	1	WGD	At1G80590
RsWRKY9	Rhsim03G0192300	IIe	365	WRKYGQK	C2H2	1	WGD	At5G52830
RsWRKY10	Rhsim03G0198800	III	382	WRKYGQK	C2HC	1	WGD	AT4G23810
RsWRKY11	Rhsim04G0007000	I	669	WRKYGQK	C2H2	2	WGD	AT5G56270
				WRKYGQK				
RsWRKY12	Rhsim04G0015000	I	595	WRKYGQK	C2H2	2	WGD	AT4G26640
RsWRKY13	Rhsim04G0052400	IIb	578	WRKYGQK	C2H2	1	WGD	At1G68150
RsWRKY14	Rhsim04G0158500	IIc	292	WRKYGQK	C2H2	1	Dispersed	AT5G64810
RsWRKY15	Rhsim04G0240600	IIc	328	WRKYGQK	C2H2	1	WGD	AT5G13080
RsWRKY16	Rhsim05G0168200	I	545	WRKYGQK	C2H2	2	Transposed	AT2G38470
				**WRK--------**				
RsWRKY17	Rhsim05G0226500	I	351	WRKYGQK	C2H2	2	WGD	AT2G38470
RsWRKY18	Rhsim05G0231400	III	330	WRKYGQK	C2HC	1	WGD	At1G66560
RsWRKY19	Rhsim05G0231500	III	410	WRKYGQK	C2HC	1	Tandem	AT5G22570
RsWRKY20	Rhsim06G0054700	IId	331	WRKYGQK	C2H2	1	WGD	At4G24240
RsWRKY21	Rhsim06G0156700	IIe	429	**WRKYGRK**	C2H2	1	Transposed	AT5G45270
RsWRKY22	Rhsim06G0164400	IIb	540	WRKYGQK	C2H2	1	WGD	At1G18860
RsWRKY23	Rhsim06G0174100	I	476	WRKYGQK	C2H2	2	Dispersed	AT4G26640
				WRKYGQK				
RsWRKY24	Rhsim06G0220000	IIb	478	WRKYGQK	C2H2	1	WGD	At1G69810
RsWRKY25	Rhsim06G0226600	I	681	WRKYGQK	C2H2	2	WGD	AT4G26640
				WRKYGQK				
RsWRKY26	Rhsim06G0235700	I	716	WRKYGQK	C2H2	2	WGD	AT5G56270
				WRKYGQK				
RsWRKY27	Rhsim07G0005900	III	327	WRKYGQK	C2HC	1	WGD	AT4G11070
RsWRKY28	Rhsim07G0009800	IIc	223	WRKYGQK	C2H2	1	WGD	AT3G01970
RsWRKY29	Rhsim07G0010600	IIe	325	WRKYGQK	C2H2	1	WGD	At1G30650
RsWRKY30	Rhsim07G0037500	IIc	125	WRKYGQK	C2H2	1	Transposed	At5G46350
RsWRKY31	Rhsim07G0083100	III	351	WRKYGQK	C2HC	1	WGD	At2G46400
RsWRKY32	Rhsim07G0087300	III	129	WRKYGQK	Lost	1	WGD	At4G18170
RsWRKY33	Rhsim07G0225700	IId	343	WRKYGQK	C2H2	1	WGD	At2G23320
RsWRKY34	Rhsim08G0072700	I	557	WRKYGQK	C2H2	2	Dispersed	AT1G13960
				WRKYGQK				
RsWRKY35	Rhsim08G0098300	IId	368	WRKYGQK	C2H2	1	WGD	At2G30590
RsWRKY36	Rhsim08G0158800	IIb	559	WRKYGQK	C2H2	1	Dispersed	At4G22070
RsWRKY37	Rhsim08G0205900	IIa	330	WRKYGQK	C2H2	1	WGD	At1G80840
RsWRKY38	Rhsim10G0005100	IIc	176	WRKYGQK	C2H2	1	Dispersed	At4G39410
RsWRKY39	Rhsim10G0147700	IIc	264	WRKYGQK	C2H2	1	Proximal	At2G47260
RsWRKY40	Rhsim11G0062500	III	227	WRKYGQK	C2HC	1	Tandem	AT5G01900
RsWRKY41	Rhsim11G0064200	III	214	**WRQCRRK**	C2HC	1	Transposed	At1G66550
RsWRKY42	Rhsim11G0120500	III	1671	WRKYGQK	Lost	1	Tandem	At5G24110
RsWRKY43	Rhsim12G0007700	IIc	325	WRKYGQK	C2H2	1	Transposed	At2G46130
RsWRKY44	Rhsim12G0077700	I	535	WRKYGQK	C2H2	2	Dispersed	AT1G13960
				WRKYGQK				
RsWRKY45	Rhsim12G0094500	IIc	184	WRKYGQK	C2H2	1	Dispersed	At2G44745
RsWRKY46	Rhsim12G0134800	IIc	471	WRKYGQK	C2H2	1	Dispersed	At5G41570
RsWRKY47	Rhsim12G0187400	IIa	331	WRKYGQK	C2H2	1	WGD	At5G15130
RsWRKY48	Rhsim13G0003300	IIc	333	WRKYGQK	C2H2	1	Dispersed	AT5G49520
RsWRKY49	Rhsim13G0061600	IId	353	WRKYGQK	C2H2	1	Transposed	AT3G04670
RsWRKY50	Rhsim13G0063200	I	562	WRKYGQK	C2H2	2	WGD	AT2G38470
				WRKYGQK				
RsWRKY51	Rhsim13G0125600	IIe	484	WRKYGQK	C2H2	1	Transposed	AT5G45050
RsWRKY52	Rhsim13G0151900	IIb	583	WRKYGQK	C2H2	1	WGD	At1G62300
RsWRKY53	Rhsim13G0187900	IIe	271	WRKYGQK	C2H2	1	Transposed	AT4G23550
RsWRKY54	RhsimUnG0056300	IIb	541	WRKYGQK	C2H2	1	Transposed	AT4G01720
RsWRKY55	RhsimUnG0080500	III	547	WRKYGQK	Lost	1	Dispersed	AT3G56400
RsWRKY56	RhsimUnG0083000	III	268	WRKYGQK	C2HC	1	Dispersed	At2G40740
RsWRKY57	RhsimUnG0199600	IIb	566	WRKYGQK	C2H2	1	Transposed	At4G04450

The values in bold show variation of conserved heptapeptide motif WRKYGQK.

### 3.2 Classification of the RsWRKY TFs and phylogenetic analysis

Except for four RsWRKY proteins (RsWRKY3, RsWRKY32, RsWRKY42, and RsWRKY55), all other RsWRKYs were categorized into 3 main groups and 7 subgroups based on structural characteristics (Supplementary Figure S1, Table 1). These four RsWRKY proteins cannot be assigned to either group since they lack a zinc-finger motif or only have a partial one.

**FIGURE 1:**
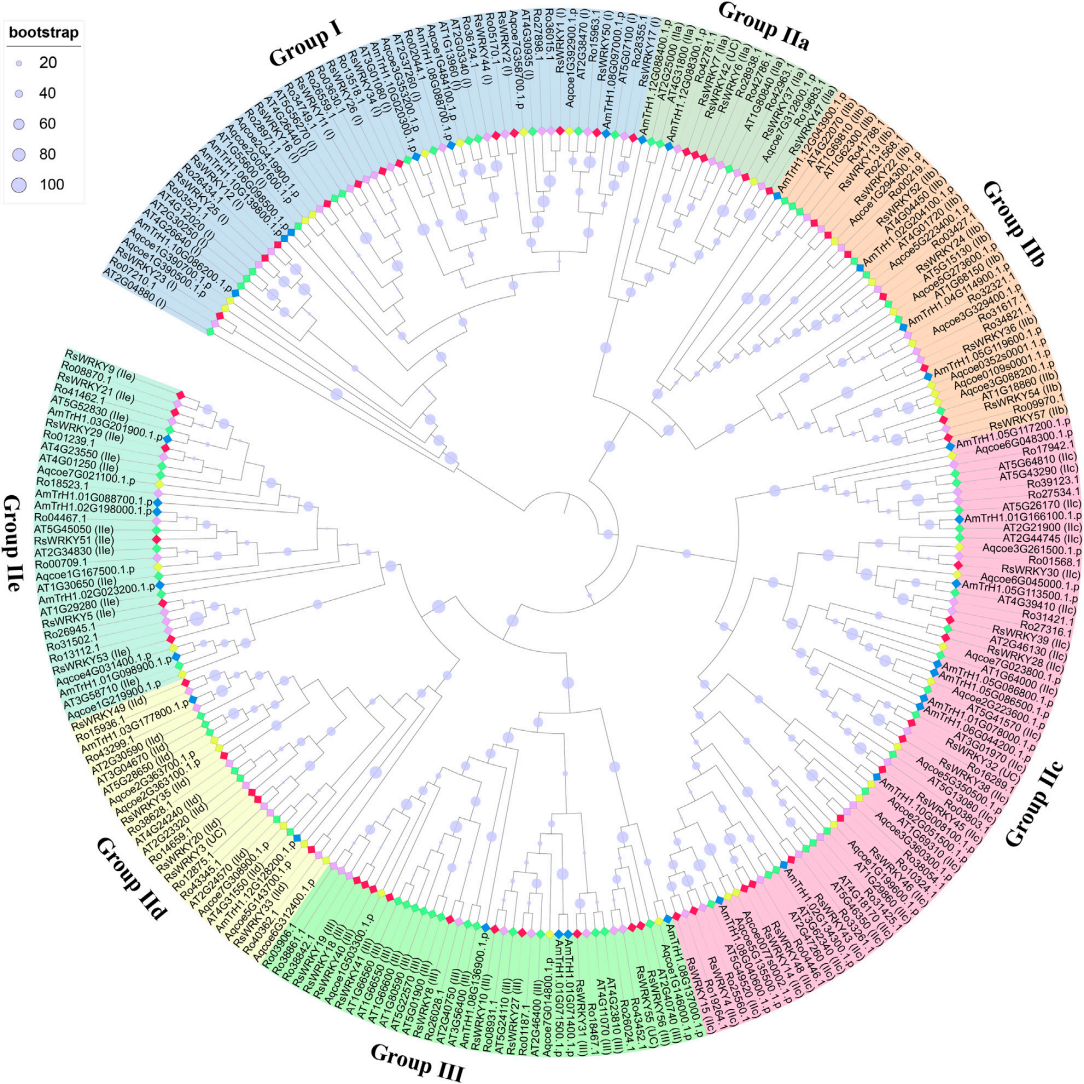
Phylogenetic analysis of WRKY proteins in azalea, *R. ovatum*, *A. thaliana*, *A. coerulea*, and *A. trichopoda*. The phylogenetic tree was constructed using maximum likelihood method based on the 268 WRKY domain sequences. The letters in parentheses that follow the names of the *RsWRKY* genes denote categorization outcomes based on structural characteristic, while the letters that follow *AtWRKY* genes expressed the previous grouping in *Arabidopsis*. The WRKY proteins from azalea, *R. ovatum*, *A. thaliana*, *A. coerulea*, and *A. trichopoda* are represented by the red, purple, green, yellow, and blue rhombuses, respectively. UC: unclassified.

In the present study, we identified 68, 39, and 32 *WRKY* genes in *R. ovatum*, *A. coerulea*, and *A. trichopoda*, respectively (Supplementary Table S2). According to the topology of the phylogenetic tree, as well as the categorization and nomenclature of *WRKY* genes in *A. thaliana*, 268 members of the WRKY family from five diverse species were divided into 3 main groups and 7 subgroups (Figure 1; Table 2). As shown in Figure 1, all groups in our study were supported by previous studies in *A. thaliana*, and all grouping outcomes in azalea based on structure characteristic were supported by the phylogenetic analysis.

**TABLE 2:** The categorization of *WRKY* genes in five species.

Species	Number of genes included							
	I	IIa	IIb	IIc	IId	IIe	III	Total
Azalea	12	5	7	12	5	6	10	57
*Rhododendron ovatum*	15	5	8	15	7	9	9	68
*Arabidopsis thaliana*	13	4	7	18	7	9	14	72
*Aquigelia coerulea*	8	1	7	11	5	4	3	39
*Amborella trichopoda*	6	2	4	10	2	4	4	32

According to the topology of the phylogenetic tree, the four unclassified members (RsWRKY3, RsWRKY 32, RsWRKY 42 and RsWRKY 55) were classified into different groups, with RsWRKY3 classified into group IId, RsWRKY 32 classified into group IIc, RsWRKY 42 classified into group IIa, and RsWRKY 55 classified into group III (Figure 1). In summary, Groups I, II, and III in azalea had 12, 35, and 10 RsWRKY members, respectively. Group II comprised of five subgroups, and each of subgroup IIa, IIb, IIc, IId, and IIe contained 5, 7, 12, 5, and 6 members, respectively.

Compared with a previous study (Wan et al., 2022), a better phylogenetic analysis and grouping outcomes was achieved in our study. For instance, the previous research mixed two different clades into group III in phylogenetic tree, which results in a categorization error. Rhsim04G0158500 (RsWRKY14) and Rhsim13G0003300 (RsWRKY48), which both have the typical “WRKYGQK” structure and CX4C structure, should both be in Group IIc but have been put in Group III in the previous research.

### 3.3 Comparative analysis of *WRKY* genes

**FIGURE 2:**
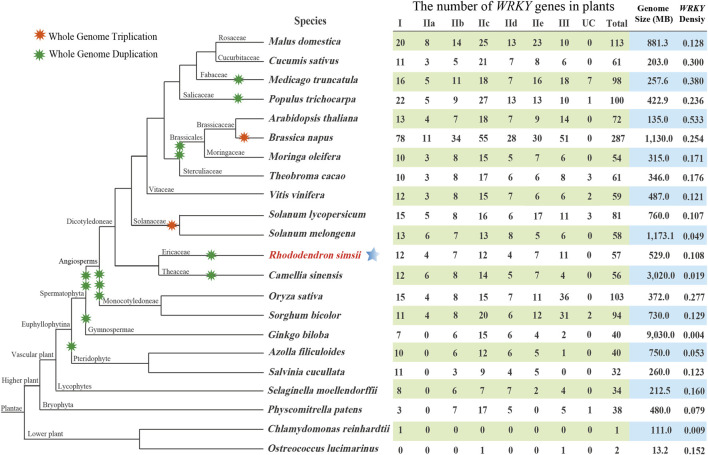
The evolutionary relationships of the 22 plant species and the specific information of the *WRKY* gene family in each genome. UC: unclassified.

To explore the evolution of the *WRKY* gene family, a comparative genomic study of *WRKY* genes was performed in 22 typical plants. Figure 2 depicts the evolutionary relationships of these species as well as the number of *WRKY* genes present in each genome. Our research revealed that *WRKY* gene numbers in lower plants are far less than that in higher plants, which indicated that the *WRKY* genes expanded considerably during the evolution from lower plant species to higher plant species. The genome size of *C. sinensis* (3020.0 MB) was larger than all other angiosperm species. However, compared to all other angiosperm plants, it possesses a genome with a reduced density of *WRKY* genes, which suggests that many *CsWRKYs* were lost during polyploid speciation. In addition, *G. biloba* exhibited an exceptionally low *WRKY* gene density compared to all other species, including lower plants. This phenomenon can be explained by the expansion of the *G. biloba* genome, which was accompanied by a significant extension of introns, mainly caused by the insertion of long terminal repeats rather than the recent occurrence of whole-genome duplication events (Liu et al., 2021).

### 3.4 Gene duplication and *Ka*/*Ks* analysis

**FIGURE 3:**
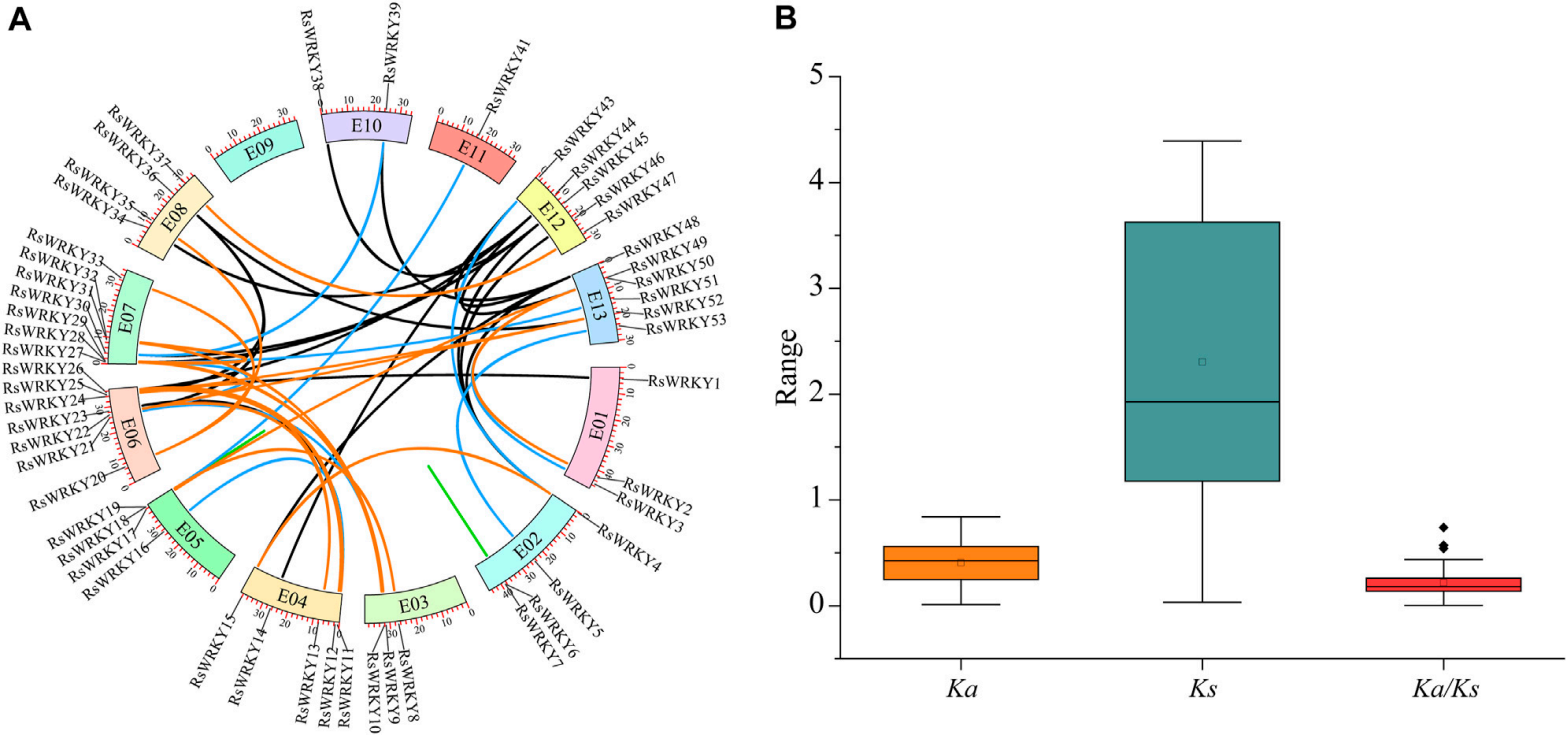
**(A)** Genomic locations of *RsWRKYs* and all duplicated gene pairs in the azalea genome. Duplicated *RsWRKY* gene pairs were indicated by the colored lines: WGD pairs (orange), tandem duplication pairs (green), transposed duplication pairs (blue), dispersed duplication pairs (black). Color boxes with a number inside it represents chromosomes. **(B)** *Ka*, *Ks*, and *Ka/Ks* ratio of all duplication gene pairs. The box plots are exhibiting the distributions of *Ka*, *Ks*, and *Ka/Ks* values. The small square and the line in the box represent average and median values of the *Ka*, *Ks*, and *Ka/Ks* values, respectively.

Using DupGen finder, the 57 *RsWRKY* genes were divided into the following five groups in the current study: 27 WGD (47.37%), 5 tandem duplication (8.77%), 1 proximal tandem duplication (1.75%), 12 transposed tandem duplication (21.05%), and 12 dispersed tandem duplication (21.05%) (Table 1; Supplementary Figure S2). 52 duplication gene pairs in all were found in *RsWRKYs* (Supplementary Table S2; Figure 3A). Then, synonymous (*Ks*) and non-synonymous (*Ka*) mutations were examined in these gene pairs (Supplementary Table S3; Figure 3B). Genes were frequently subjected to purifying selection (*Ka*/*Ks* < 1), positive selection (*Ka*/*Ks* > 1), and neutral selection (*Ka*/*Ks* = 1) during the course of evolution. In the current study, all 52 *RsWRKY* duplication gene pairs showed *Ka*/*Ks* rates lower than 1, which suggests that purifying selection and less divergence occurred among them.

**FIGURE 4:**
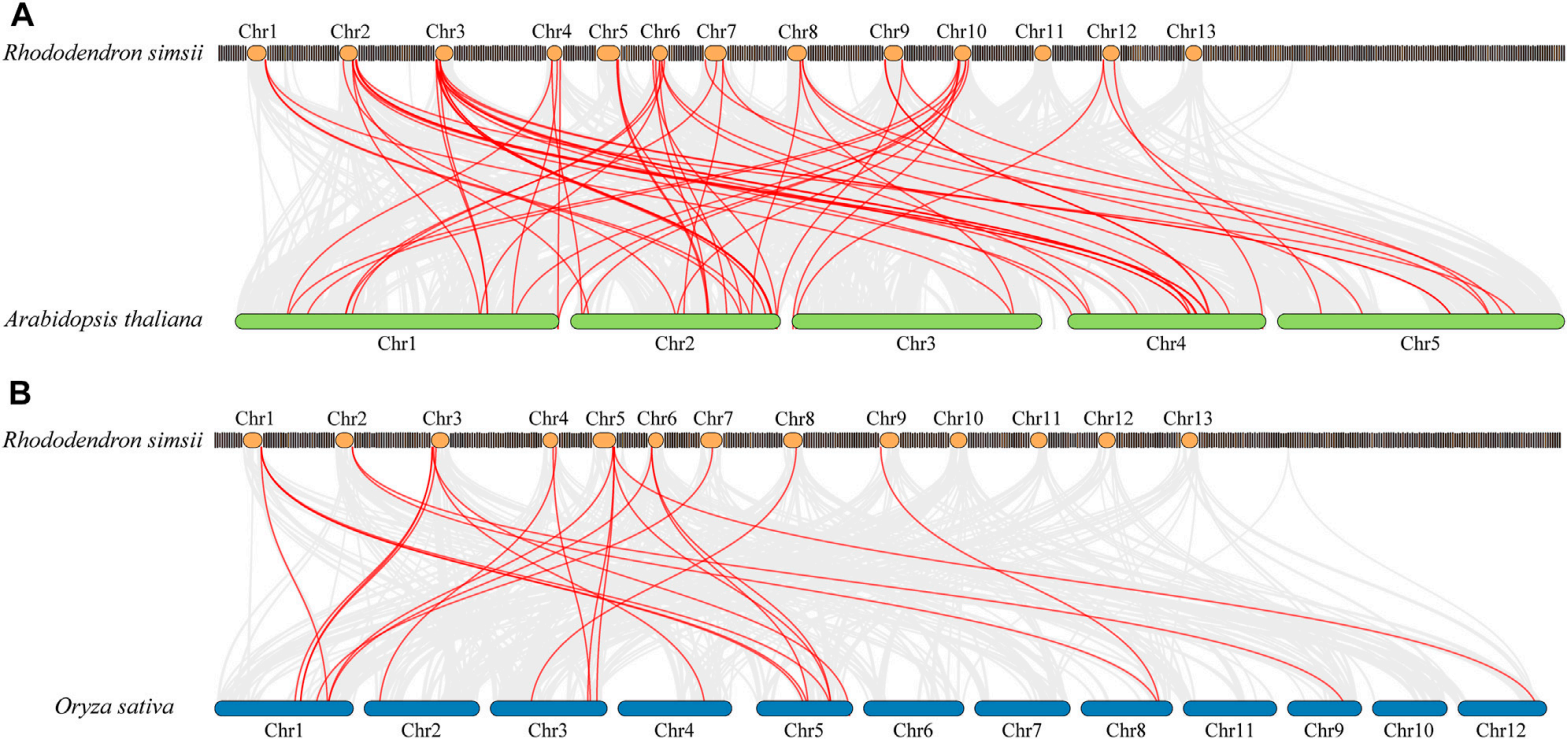
Comparative physical mapping showing the orthologous relationships of *RsWRKY* genes with *Arabidopsis* **(A)** and rice **(B)**.

In order to explore the potential evolutionary processes of the *WRKY* gene family, collinearity analysis was employed to examine the orthologous relationships of *WRKY* family genes in azalea, *Arabidopsis*, and rice (Figure 4). A total of 63 pairs of orthologs were discovered between azalea and *Arabidopsis* (Supplementary Table S4), while 24 pairs of orthologs were found between azalea and rice (Supplementary Table S5), these *RsWRKY* genes and the corresponding *AtWRKY* or *OsWRKY* genes shared a common ancestor. Some ortholog relationships involved one *RsWRKY* corresponding to one ortholog gene (e.g., *RsWRKY20*-AT2G23320 and *RsWRKY18*-LOC_Os05g25770), while others had one *RsWRKY* corresponding to multiple ortholog genes (e.g., *RsWRKY22*-AT1G62300/AT4G22070/AT4G04450 and *RsWRKY17*-LOC_Os01g61080/LOC_Os05g39720/LOC_Os05g27730). These results suggest that duplication events have played a significant role in the evolution and functional diversification of the *WRKY* family.

### 3.5 Expression profiling of *RsWRKY* genes in three azalea varieties

**FIGURE 5:**
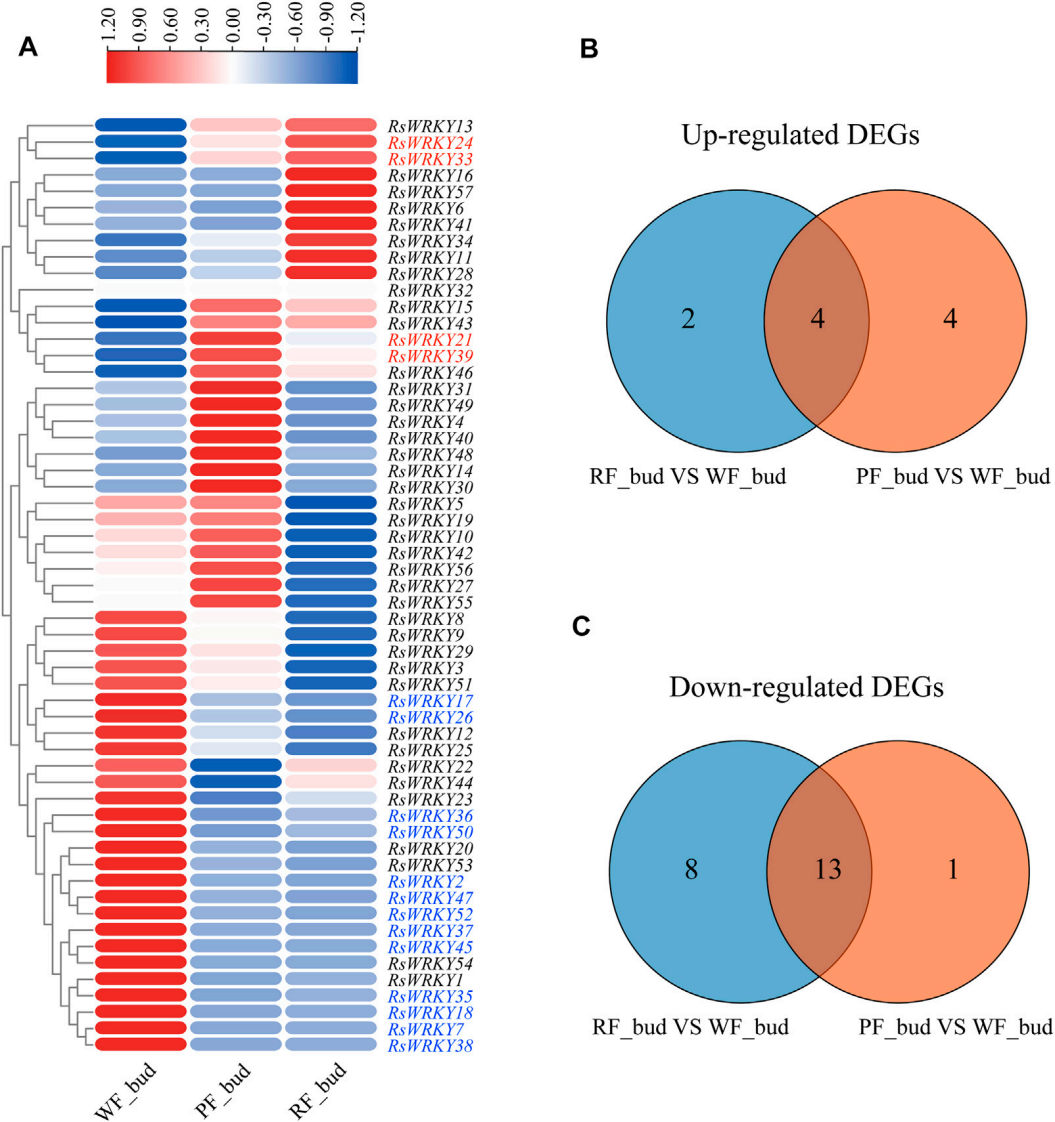
Identification of candidate *RsWRKYs* associated with anthocyanin biosynthesis among white flowering (WF), red flowering (RF) and pink flowering (PF) azaleas at the bud stage. **(A)** Heatmap of *RsWRKYs* at the full bloom stage, the scale bar represents relative expression value. **(B)** Venn diagram indicates the unique and common upregulated DEGs are found in set “RF_bud VS. WF_bud” and set “PF_bud VS. WF_bud.” **(C)** Venn diagram indicates the unique and common downregulated DEGs are found in set “RF_bud VS. WF_bud” and set “PF_bud VS. WF_bud.”

**FIGURE 6:**
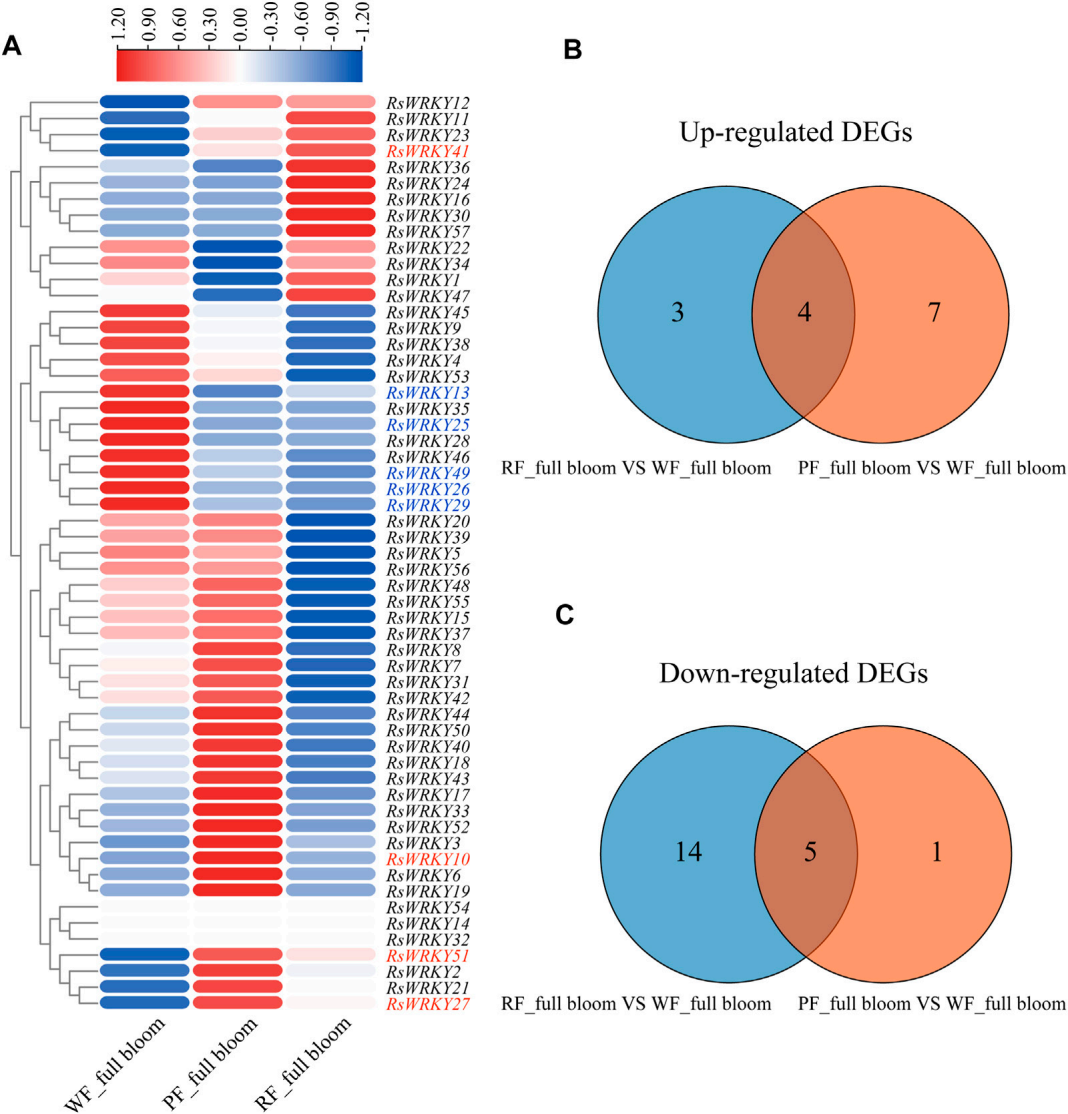
Identification of candidate *RsWRKYs* associated with anthocyanin biosynthesis among white flowering (WF), red flowering (RF) and pink flowering (PF) azaleas at the full bloom stage. **(A)** Heatmap of *RsWRKYs* at the full bloom stage, the scale bar represents relative expression value. **(B)** Venn diagram indicates the unique and common upregulated DEGs are found in set “RF_ full bloom VS. WF_ full bloom” and set “PF_ full bloom VS. WF_ full bloom.” **(C)** Venn diagram indicates the unique and common downregulated DEGs are found in set “RF_ full bloom VS. WF_ full bloom” and set “PF_ full bloom VS. WF_ full bloom.”

The expression profiles of *RsWRKY* genes in three azalea cultivars with different flower colors were analyzed using RNA-seq data to identify *WRKY* genes involved in anthocyanin biosynthesis in azaleas (Figures 5, 6; Supplementary Tables S6, S7). Genes that showed absolute |fold change| ≥ 2 (log2 |fold change| ≥ 1) for differential expression between two azalea varieties were defined as differentially expressed genes (DEGs). As a result, there are 6 upregulated DEGs and 21 downregulated DEGs between red flower (RF) and white flower (WF) azaleas at the bud stage. In addition, 8 upregulated DEGs and 14 downregulated DEGs were identified between pink flower (PF) and white flower (WF) azaleas at the bud stage. Combined with the two DEG sets ((WF versus RF) and (WF versus PF)), the candidate *RsWRKYs* associated with anthocyanin biosynthesis were screened. The expression level of 4 DEGs (*RsWRKY21*, *24*, *33* and *39*) was upregulated in colored-flower azaleas (PF and RF), indicating that these genes might play a role in the positive control of anthocyanin production at the bud stage. While the expression level of 13 DEGs (*RsWRKY2*, *7*, *17*, *18*, *26*, *35*, *36*, *37*, *38*, *45*, *47*, *50* and *52*) was downregulated in colored-flower azaleas (PF and RF), indicating that these genes may have a role in suppressing the synthesis of anthocyanins at the bud stage (Figure 5; Supplementary Table S6). Using the same comparative strategy, we identified 4 candidate genes (*RsWRKY10*, *27*, *41* and *51*) that might play a role in the positive control of anthocyanin production at the full bloom stage, and 5 candidate genes (*RsWRKY13*, *25*, *26*, *29* and *49*) that might be involved in negatively regulating the anthocyanin biosynthesis at the full bloom stage (Figure 6; Supplementary Table S7).

### 3.6 Validation of RNA-seq-based gene expression

**FIGURE 7:**
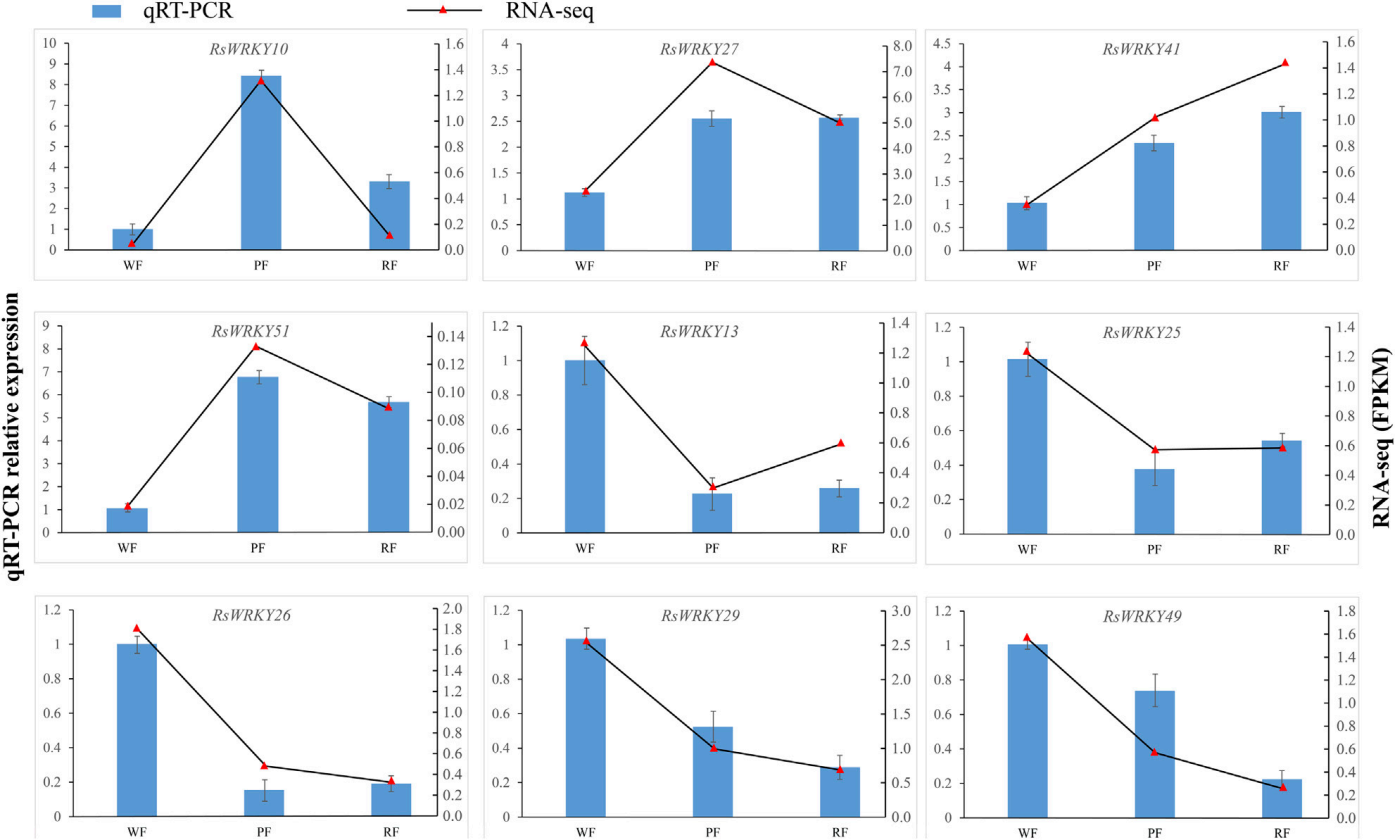
qRT-PCR validation of 9 candidate *RsWRKY* genes at the full bloom stage. WF, PF, and RF represent white-flowering variety, pink-flowering variety, the red-flowering variety, respectively.

To confirm the accuracy of the RNA-seq results, 9 candidate genes related to anthocyanin biosynthesis at the full bloom stage were chosen and examined using qRT-PCR. In RT-PCR tests, the genes that were up- or downregulated in RNA-seq experiments were likewise up- or downregulated. Even though the values of the gene fold change differ between the RNA-seq and RT-PCR data, the expression trend of gene is the same. For instance, RNA-seq data revealed that the expression of *RsWRKY10* was 19.8 and 2 times higher in red and pink flowers than in white flowers, respectively, in RT-PCR tests, the corresponding fold change values were 8.4 and 3.3. According to RNA-seq data, the expression of *RsWRKY29* in red and pink flowers was 0.38 and 0.26 less than that of white flowers, whereas the corresponding values in RT-PCR tests were 0.52 and 0.29. In conclusion, RT-PCR results agree well with the RNA-Seq data in terms of expression trends (Supplementary Table S7; Figure 7).

### 3.7 GO enrichment analysis of candidate genes

**FIGURE 8:**
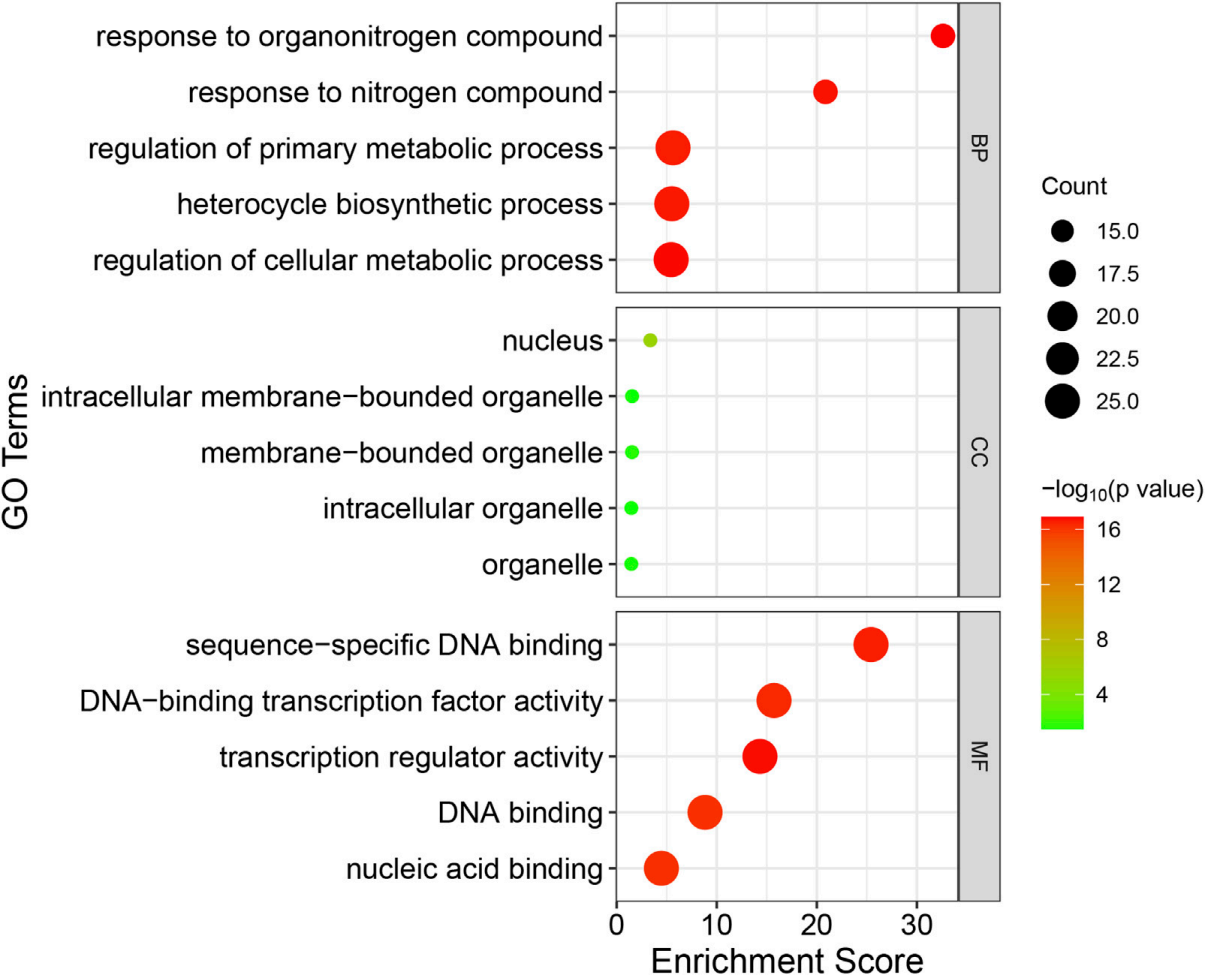
GO enrichment analysis of the candidate *RsWRKY* genes. GO was performed with three main categories: molecular function (MF), cellular component (CC) and biological process (BP). GO terms with *p*-value < 0.05 were identified as significant.

In order to have a comprehensive understanding of the biological function of the candidate genes, gene ontology (GO) annotation and enrichment analysis of the 25 candidate *RsWRKY* genes were performed in present study (Figure 8; Supplementary Table S8). 123 GO terms showed significant enrichment, with 9 representing molecular functions, 5 representing cellular components, and 109 representing biological processes. In the molecular function category, “transcription regulator activity,” “sequence-specific DNA binding” and “DNA-binding transcription factor activity” were the most enriched terms. In the cellular component category, “nucleus,” “intracellular membrane-bounded organelle” and “membrane-bounded organelle” were the most enriched. In the biological process category, “response to organonitrogen compound,” “regulation of cellular metabolic process,” “response to nitrogen compound” and “gene expression” were the most enriched terms. GO enrichment results indicate that the candidate RsWRKY TF has primarily functions centered around common transcription factor functions such as regulation of gene expression and transcription regulatory.

### 3.8 Three-dimensional structure prediction of candidate genes

**FIGURE 9:**
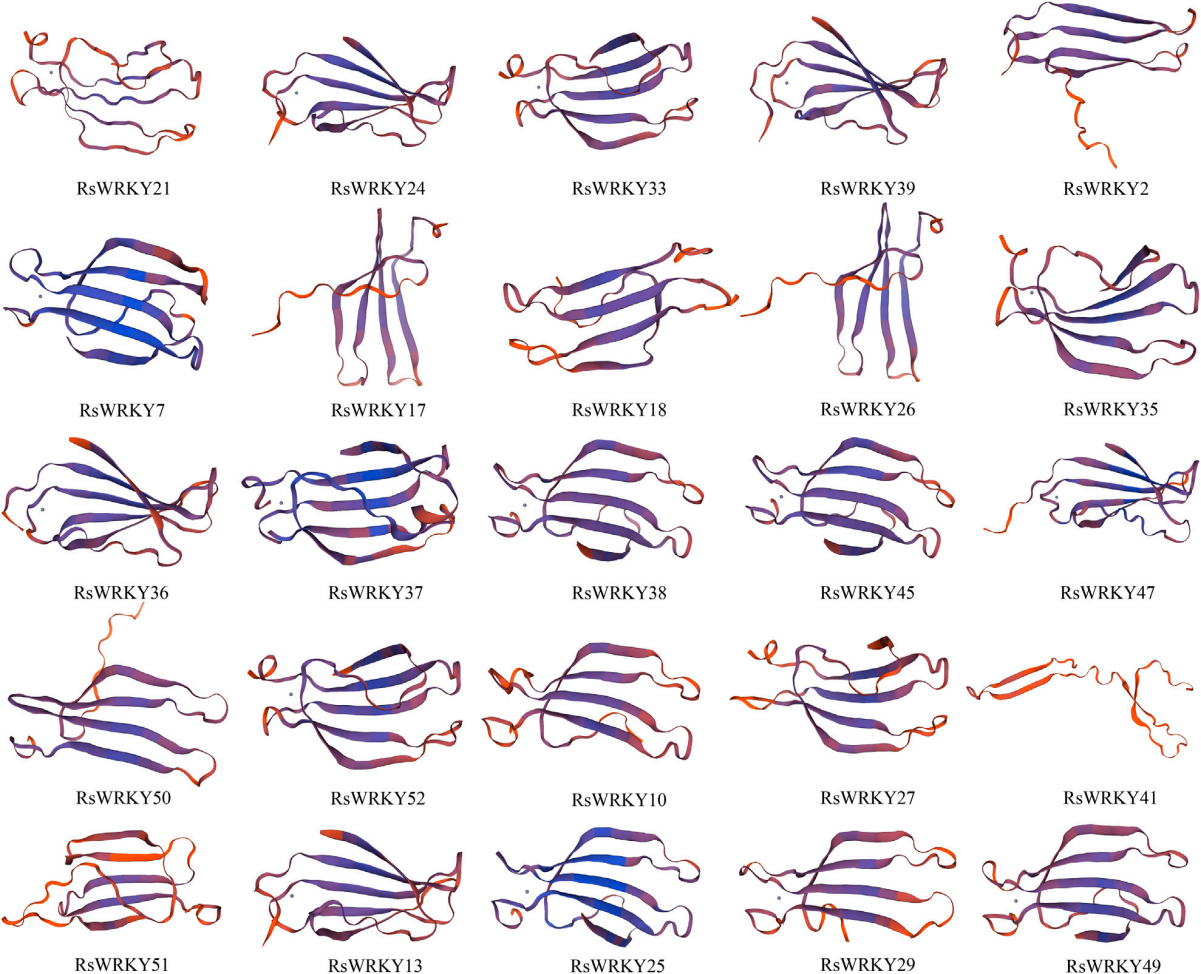
Three-dimensional (3D) protein structures of 25 candidate RSWRKYs. Broad strips are β-sheets and dots are zinc ions.

We utilized SWISS-Model to predict the 3D protein structures of 25 candidate RsWRKYs (Figure 9). Out of the 25 models, 25 were successfully defined by at least 30% identity of the target to the template, which is widely accepted as a threshold for successful modeling (Xiang, 2006). The majority of QMEANDisCo global values were higher than 0.50, indicating good quality models, except for RsWRKY18 and RsWRKY41, which had QMEANDisCo global values of 0.49 and 0.44, respectively, indicating lower quality models (Supplementary Table S9). The GMQE values ranged from 0.05 to 0.27, which were very low because the models covered only WRKY domain sequences of the target proteins. All 25 models were monomer oligo-state, and different 3D structures were observed in RsWRKY41. Tertiary structure prediction results indicated that most RsWRKYs contained a zinc ion and the RsWRKY proteins domain were mainly composed of β folds.

## 4 Discussion

*WRKY* genes are a vast family of TFs that are present in almost all plant species and serve key roles in a variety of physiological functions (Rinerson et al., 2015; Li W. et al., 2020; Wani et al., 2021). Numerous plant species have had their WRKY TFs identified at the entire genome level (Guo et al., 2014; He et al., 2016; Kumar et al., 2016; Wang et al., 2019; Chen et al., 2020b; Qin et al., 2022). In this research, we reported a genome-wide analysis of the *WRKY* family in azalea and 57 *RsWRKY* genes was identified. The WRKY domain is the crucial element that controls how the WRKY protein binds specifically to the cis-element W-box. According to sequence alignment analysis, the majority of RsWRKY proteins shared exceptionally heptapeptide motif WRKYGQK at their N-termini. But this analysis also uncovered other variants, including WRKYGEK (RsWRKY2), WRKYGRK (RsWRKY21), and WRQCRRK (RsWRKY41) (Supplementary Figure S1). The *Arabidopsis* (Eulgem et al., 2000), maize (Hu et al., 2021), apple (Qin et al., 2022), carrot (*Daucus carota* subsp. *sativus* L.) (Nan and Gao, 2019), and duckweed (*Spirodela polyrhiza* L.) (Zhao et al., 2021) WRKY members have also shown this occurrence. Additionally, it was revealed that one protein (RsWRKY16) possessing an incomplete WRKYGQK sequence and four RsWRKY proteins (RsWRKY3, 32, 42 and 55) that lacked the entire zinc finger motif were also identified as RsWRKY family members. Previous studies have demonstrated that the presence of both the heptapeptide motif WRKYGQK and the zinc finger motif is necessary for the WRKY TFs' significant binding affinity to their homologous cis-acting W-box element (TTGACC/T) (Wani et al., 2021). Due to this, modifications to the heptapeptide motif and the absence of the zinc finger motif may impact the regular interactions of RsWRKYs with their downstream target genes. Therefore, it may be essential to further explore the role and binding specificity of these eight RsWRKY proteins.

An ML phylogenetic tree based on conserved WRKY domains was constructed in this study using the WRKY proteins from azalea, *R. ovatum* (a related asteroid eudicot), *Arabidopsis* (a rosid eudicot), *A. coerulea* (a basal eudicot), and *A. trichopoda* (a basal angiosperm). As shown in Figure 1, the RsWRKY proteins from Groups IIa and IIb were more closely related, while Groups IId and IIe had a close relationship. Since Groups IIa and IIb and Groups IId and IIe split off from the progenitor of terrestrial plants considerably later than the other Groups (Zhang and Wang, 2005), it was assumed that these two Groups would merge to form two new subfamilies, IIa + b and IId + e, respectively (Rushton et al., 2010; Chen et al., 2017). We investigated the *WRKY* gene family in azalea and 21 other species, including 19 higher plants and 2 lower plants. Comparative genomic analysis revealed that single-celled *C. reinhardtii* and *O. lucimarinus* have 1 and 2 *WRKY* genes respectively. The moss *P. patens*' genome, however, encodes up to 38 *WRKY* members (Figure 2), showing a substantial increase following this species diverged from green algae. *P. patens* has a role in evolutionary analysis, which makes it possible to compare genomes with aquatic algae and vascular plants in order to reconstruct evolutionary changes brought on by land conquest (Rensing et al., 2008). The growth of the WRKY family in *P. patens* illustrates the significance of WRKY proteins in numerous physiological processes, particularly the complex functions held by higher plants. Furthermore, *G. biloba* had fewer *WRKY* genes than most species while having a larger genome than all other species, proving that genome size is not correlated with the amount of *WRKY* genes.

Gene duplication events are crucial in the rapid expansion and evolution of gene families (Maher et al., 2006). The most significant factors in the growth of gene families during evolution are WGD and tandem duplication events (Wang et al., 2012). Many transcription factor gene families, such as MADS-box, bHLH, and bZIP, most likely expanded through WGD duplication (Liu et al., 2014; Wang B. et al., 2022; Wang X. J. et al., 2022). While other transcription factor gene families, such as NBS-LRR and ERF, were expanded as a result of tandem duplication (Liu Z. et al., 2019; Li P. et al., 2020; Zhong et al., 2022). Since 47.37% of the *RsWRKY* genes were duplicated and retained through WGD duplication, our research suggested that WGD duplication was the major causes for the expansion of the *RsWRKY* family of genes in azalea. This outcome was consistent with previous research on apple (Qin et al., 2022), carrot (Nan and Gao, 2019), walnut (*Juglans regia* L.) (Hao et al., 2021), and potato (Zhang et al., 2017). Pseudogenization (loss of function), subfunctionalization, and neofunctionalization are possible outcomes of duplicated genes (Lynch and Conery, 2000). The WGD duplicated gene pair *RsWRKY24*/*RsWRKY52* from Group IIb with varied levels of expression may be due to the fact that duplicated genes can be stably maintained if they differ in some aspects of their functions during selection and evolution. We also evaluated the rate of synonymous (*Ks*) to non-synonymous (*Ka*) mutation for all duplication gene pairs. It is well known that genes are generally subjected to three different types of selection—purifying selection (*Ka*/*Ks <* 1), positive selection (*Ka*/*Ks* > 1), and neutral selection (*Ka*/*Ks* = 1) (Hurst, 2002). All of the *RsWRKY* duplication gene pairs in our study had *Ka*/*Ks* ratios smaller than 1, indicating that they had undergone strong Darwinian purifying positive selection. Genomic comparison is an effective way to transfer knowledge about the genome of a well-studied taxonomic group to a less studied one (Lyons et al., 2008). In our research, we discovered that 63 pairs of *R. simsii* and *Arabidopsis WRKY* genes exhibit a collinear relationship, while 24 pairs of *WRKY* genes show a collinear relationship between *R. simsii* and rice (Supplementary Table S4, S5). This finding suggests that these gene pairs are orthologous, have common ancestors, and have been preserved throughout evolution, potentially performing similar functions. The identification of *WRKY* genes in *Arabidopsis* and rice serves as a reference for future studies on *RsWRKY* genes.

Azaleas are well-known decorative plants with major cultural and economic significance. Flower color is the primary trait in the breeding and creation of new ornamental cultivars. *WRKY* genes have been shown to be involved in the control of the anthocyanin biosynthesis (see “Introduction” section), and are thus key factors for the breeding of azaleas. In this study, we discovered 17 candidate *RsWRKY* genes that may be involved in the synthesis of anthocyanins at the bud stage and 9 candidate *RsWRKY* genes that may be involved in the synthesis of anthocyanins at the full bloom stage. Unexpectedly, there is little overlap between these two sets of genes (only *RsWRKY26*), which indicating that various *RsWRKY* genes are engaged in anthocyanin biosynthesis at various times of flowering. In this study, we utilized GO annotation and enrichment analysis to elucidate the functions of the potential *RsWRKY* genes, which are involved in sequence-specific DNA binding, biological process regulation, gene expression regulation, among others. However, our results solely revealed fundamental transcription factor functions, and did not provide any information related to anthocyanin synthesis. The main reason for this limitation is the insufficient research, particularly in terms of confirmed experimental investigations, on the role of *WRKY* in anthocyanin biosynthesis. As a result, further research is imperative to validate the candidate *RsWRKY* genes that were identified in this study to be associated with anthocyanin production.

## 5 Conclusion

In summary, we systematically identified and classified 57 *WRKY* genes from the *R. simsii* genome, revealing their structural and phylogenetic characteristics. Our comparative genomic analysis suggests that the *WRKY* gene family has significantly expanded during plant evolution, primarily through WGD duplication, and that selective pressure analysis indicates strong purifying selection during evolution. Synteny analysis further confirmed the conservation of *RsWRKY* genes with orthologs in *A. thaliana* and *O. sativa*. Additionally, we conducted expression profiling analysis of *RsWRKY* genes in three azalea varieties, providing a valuable resource for future investigation into the molecular control of anthocyanin synthesis in azalea. While further studies are needed to fully understand the functional mechanisms of candidate *RsWRKY* genes, our comprehensive analysis provides a basis for investigating the molecular mechanisms of anthocyanin biosynthesis in *Rhododendron* species and lay the foundation for future functional studies of *WRKY* genes.

## Data Availability

The original contributions presented in the study are included in the article/Supplementary Material, further inquiries can be directed to the corresponding author.
