# Supplementary material for: Genome-wide identification and bioinformatics analysis of the WRKY transcription factors and screening of candidate genes for anthocyanin biosynthesis in azalea (Rhododendron simsii)
Source: Front Genet. 2023 May 10;14:1172321. doi: 10.3389/fgene.2023.1172321 (PMC10206045; doi:10.3389/fgene.2023.1172321)
Supplement: Supplementary file 1 [file DataSheet1.docx]

Supplementary Material

Genome-wide identification and characterization of RsWRKY transcription factors related to anthocyanin biosynthesis in azalea (*Rhododendron simsii*)

**Cheng Wang ^1^, Dan Ye ^1^, Yan Li ^2^, Peiling Hu ^1^, Run Xu ^1^ and Xiaojing Wang ^3,^ ***

*** Correspondence:** Xiaojing Wang: xjwang8@gzu.edu.cn

Supplementary Figures

**
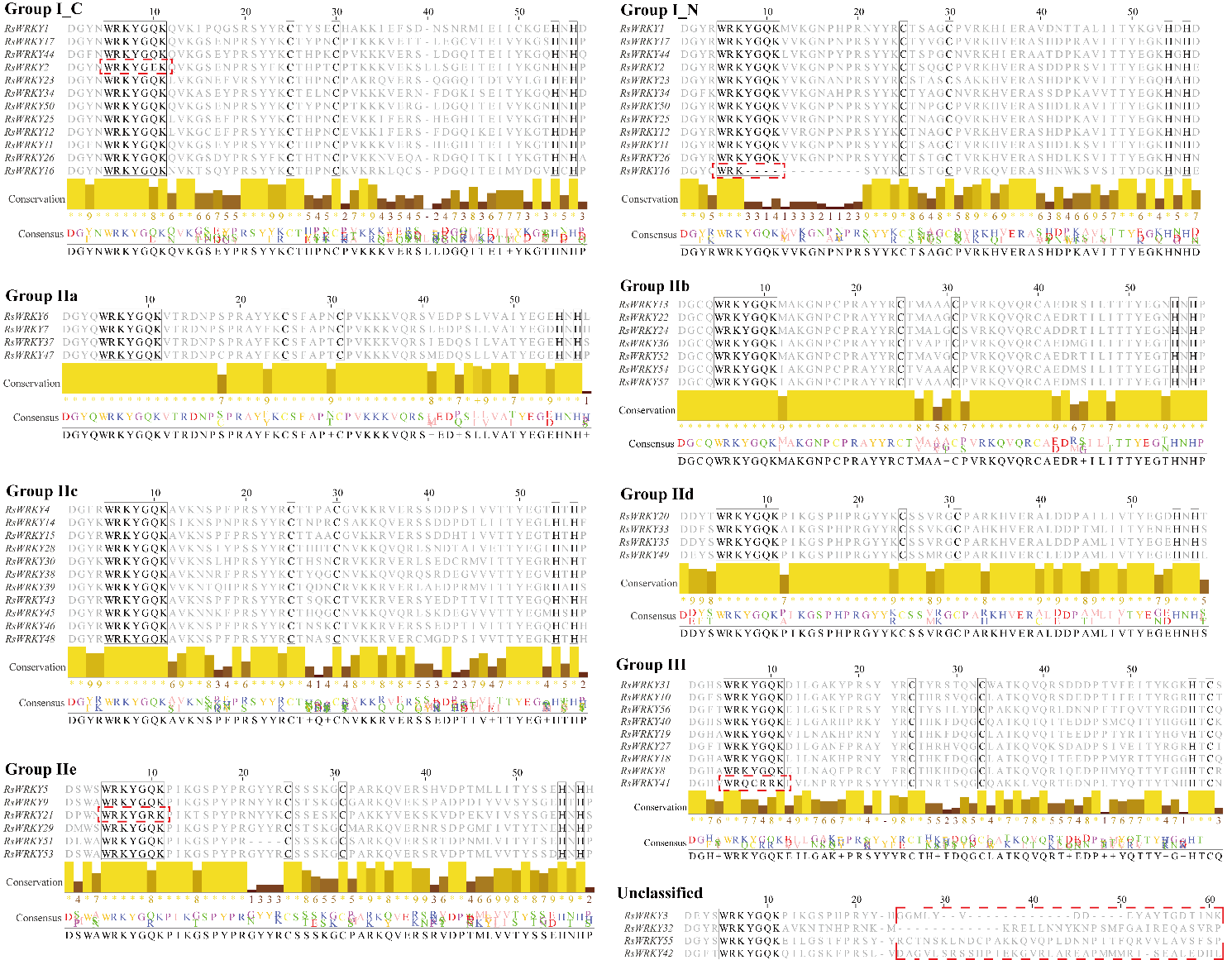
**

**Figure S1.** Multiple sequence alignment of the WRKY domain from RsWRKY protins. Black boxes indicated the highly conserved WRKYGQK and the zinc-finger motif sequences. Red boxes indicated the exceptional situations.


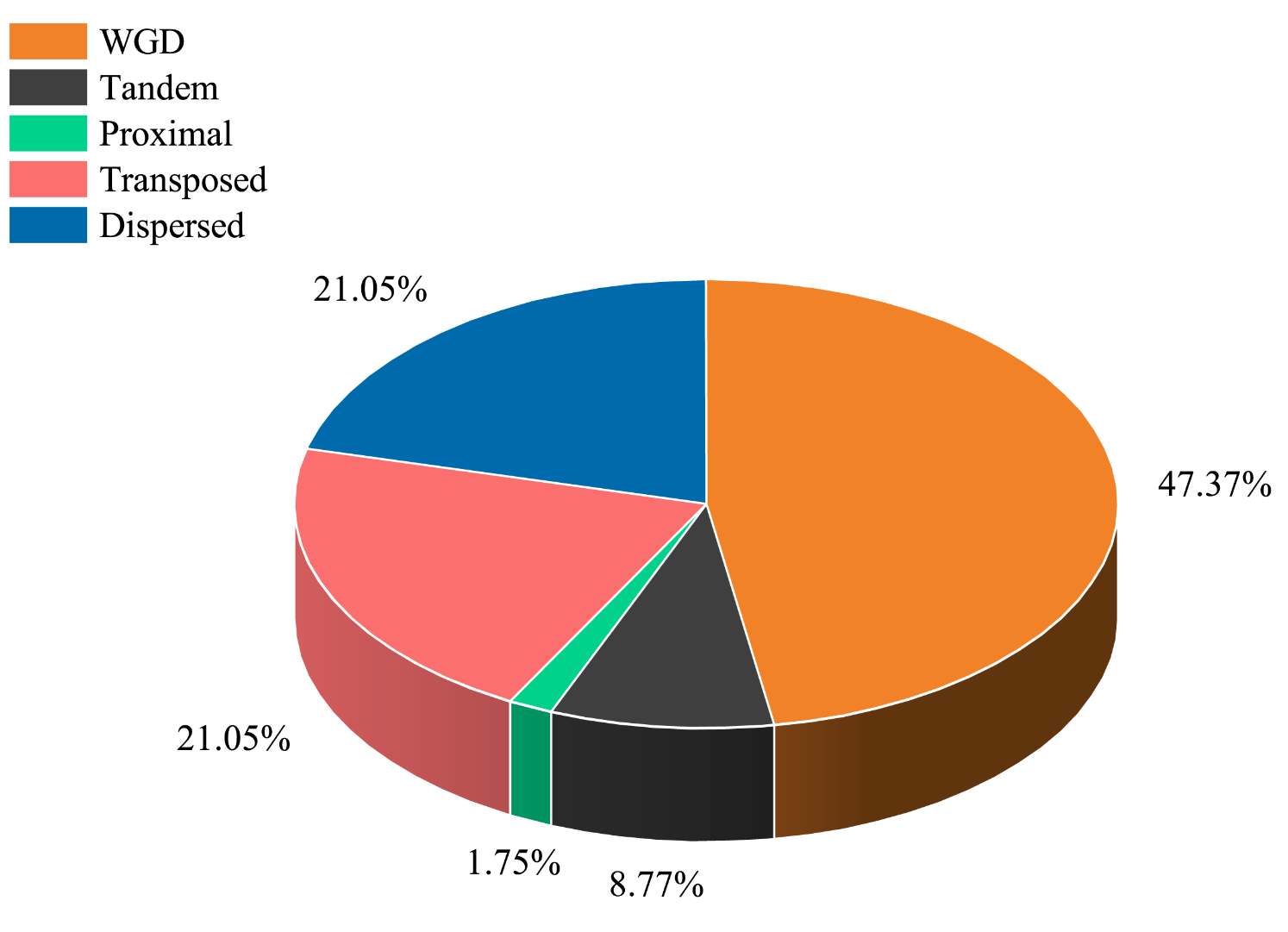


**Figure S2.** Percentage of *RsWRKYs* that come from various replication events.

**Supplementary Table:**

Table S1: The primer sequences used for qRT-PCR.

| **Gene ID** | **Forward primer** | **Reverse primer** |
| --- | --- | --- |
| *RsWRKY10* | TCTTCTTCTTCTTCTTCCT | TGTTGATGGTCTCCTAAG |
| *RsWRKY27* | GCATAATGGAGGAGACTT | CGAACACTGAGAGGAATT |
| *RsWRKY41* | TACTAACAGGACTTCTCA | TGTGTATGTGGTCTGATA |
| *RsWRKY51* | AACAACAGTAATACCAATTCAC | GCTCTTCTTCCTCTTCTTC |
| *RsWRKY13* | ACAAGGAGTGCCAAATAC | TCATCATCATCATCGTCATC |
| *RsWRKY25* | AACGGAACTAATGATGGT | GCGAATAGGCTTAACTTG |
| *RsWRKY26* | ATGCTCCAAGGTTGAATAC | AGGCTGAATCGTAATGTAAG |
| *RsWRKY29* | ACCAATACTTCTACTACTGCTAT | TCTGCTGGAACTTCACAA |
| *RsWRKY49* | CTATAAGTGTAGCAGCAT | TCTCCTTCATAAGTAACAAT |
| *RsGAPDH (Rhsim12G0106200)* | CCACAGGAACCCAGAAGAAA | CTCCTCCTTGATAGCCTCTTTG |
| *RsEF1α (Rhsim02G0008200)* | GGAACTTCATCTCACCTCCTAAC | AGAACCTGACCAACCAATCTATC |

Table S2. The *WRKY* genes i*n Rhododendron ovatum*, *Aquigelia coerulea* and *Amborella trichopoda.*

| ***Rhododendron ovatum*** | ***Aquigelia coerulea*** | ***Amborella trichopoda*** |
| --- | --- | --- |
| Ro36124.1 | Aqcoe1G390500.1.p | AmTrH1.08G088700.1.p |
| Ro02044.1 | Aqcoe3G353200.1.p | AmTrH1.08G097000.1.p |
| Ro15963.1 | Aqcoe1G392900.1.p | AmTrH1.06G098500.1.p |
| Ro26559.1 | Aqcoe1G484100.1.p | AmTrH1.10G139800.1.p |
| Ro34749.1 | Aqcoe1G390700.1.p | AmTrH1.10G020300.1.p |
| Ro05170.1 | Aqcoe2G419900.1.p | AmTrH1.10G086200.1.p |
| Ro13518.1 | Aqcoe7G358700.1.p | AmTrH1.03G177800.1.p |
| Ro03630.1 | Aqcoe2G051600.1.p | AmTrH1.12G128200.1.p |
| Ro03521.1 | Aqcoe1G146000.1.p | AmTrH1.01G078000.1.p |
| Ro28355.1 | Aqcoe7G014800.1.p | AmTrH1.01G071500.1.p |
| Ro07210.1 | Aqcoe7G308900.1.p | AmTrH1.02G134300.1.p |
| Ro26434.1 | Aqcoe6G312400.1.p | AmTrH1.01G071400.1.p |
| Ro27898.1 | Aqcoe5G350500.1.p | AmTrH1.06G044200.1.p |
| Ro36015.1 | Aqcoe2G223600.1.p | AmTrH1.01G088700.1.p |
| Ro28971.1 | Aqcoe2G363700.1.p | AmTrH1.02G023200.1.p |
| Ro18467.1 | Aqcoe2G363100.1.p | AmTrH1.10G008100.1.p |
| Ro08931.1 | Aqcoe5G135500.1.p | AmTrH1.08G040600.1.p |
| Ro25560.1 | Aqcoe0077s0002.1.p | AmTrH1.01G098900.1.p |
| Ro26024.1 | Aqcoe7G021100.1.p | AmTrH1.12G043900.1.p |
| Ro19264.1 | Aqcoe1G167500.1.p | AmTrH1.05G113500.1.p |
| Ro43299.1 | Aqcoe7G023800.1.p | AmTrH1.04G114900.1.p |
| Ro31425.1 | Aqcoe5G143700.1.p | AmTrH1.08G137000.1.p |
| Ro01187.1 | Aqcoe1G503300.1.p | AmTrH1.08G136900.1.p |
| Ro15936.1 | Aqcoe5G273600.1.p | AmTrH1.02G204100.1.p |
| Ro03803.1 | Aqcoe6G045000.1.p | AmTrH1.05G119600.1.p |
| Ro38628.1 | Aqcoe1G199600.1.p | AmTrH1.01G166100.1.p |
| Ro33261.1 | Aqcoe5G223400.1.p | AmTrH1.05G117200.1.p |
| Ro16289.1 | Aqcoe3G261500.1.p | AmTrH1.12G088300.1.p |
| Ro04446.1 | Aqcoe4G031400.1.p | AmTrH1.12G088400.1.p |
| Ro38842.1 | Aqcoe1G294300.1.p | AmTrH1.03G201900.1.p |
| Ro01239.1 | Aqcoe1G219900.1.p | AmTrH1.05G086500.1.p |
| Ro03906.1 | Aqcoe3G088200.1.p | AmTrH1.05G086800.1.p |
| Ro26945.1 | Aqcoe3G360300.1.p |  |
| Ro31502.1 | Aqcoe6G048300.1.p |  |
| Ro00709.1 | Aqcoe3G329400.1.p |  |
| Ro14659.1 | Aqcoe7G312800.1.p |  |
| Ro43452.1 | Aqcoe2G051500.1.p |  |
| Ro38054.1 | Aqcoe0352s0001.1.p |  |
| Ro10324.1 | Aqcoe0109s0001.1.p |  |
| Ro18523.1 |  |  |
| Ro40362.1 |  |  |
| Ro08870.1 |  |  |
| Ro38861.1 |  |  |
| Ro31421.1 |  |  |
| Ro27316.1 |  |  |
| Ro42786.1 |  |  |
| Ro21568.1 |  |  |
| Ro03427.1 |  |  |
| Ro01568.1 |  |  |
| Ro32321.1 |  |  |
| Ro41788.1 |  |  |
| Ro00219.1 |  |  |
| Ro09970.1 |  |  |
| Ro31617.1 |  |  |
| Ro34821.1 |  |  |
| Ro04467.1 |  |  |
| Ro27534.1 |  |  |
| Ro39123.1 |  |  |
| Ro12875.1 |  |  |
| Ro19683.1 |  |  |
| Ro42963.1 |  |  |
| Ro26028.1 |  |  |
| Ro42781.1 |  |  |
| Ro17942.1 |  |  |
| Ro41462.1 |  |  |
| Ro43345.1 |  |  |
| Ro28938.1 |  |  |
| Ro13112.1 |  |  |

Table S3. Gene duplications of *WRKY* genes in *R.simsii* with outlier *Ka/Ks* values.

| GenePair | GeneDuplication | *Ka* | *Ks* | *Ka/Ks* |
| --- | --- | --- | --- | --- |
| RsWRKY2-RsWRKY50 | WGD | 0.174313 | 0.767453 | 0.227132 |
| RsWRKY4-RsWRKY15 | WGD | 0.132522 | 1.17816 | 0.112482 |
| RsWRKY8-RsWRKY18 | WGD | 0.499356 | 3.68618 | 0.135467 |
| RsWRKY9-RsWRKY29 | WGD | 0.558275 | 2.54891 | 0.219025 |
| RsWRKY10-RsWRKY27 | WGD | 0.44274 | 1.97363 | 0.224328 |
| RsWRKY10-RsWRKY31 | WGD | 0.389616 | 1.56289 | 0.249293 |
| RsWRKY11-RsWRKY26 | WGD | 0.199293 | 0.766915 | 0.259863 |
| RsWRKY12-RsWRKY25 | WGD | 0.200614 | 0.696576 | 0.288 |
| RsWRKY13-RsWRKY22 | WGD | 0.225193 | 2.21587 | 0.101627 |
| RsWRKY13-RsWRKY24 | WGD | 0.428757 | 1.66258 | 0.257886 |
| RsWRKY17-RsWRKY50 | WGD | 0.232499 | 1.70222 | 0.136586 |
| RsWRKY20-RsWRKY33 | WGD | 0.310459 | 2.15286 | 0.144208 |
| RsWRKY20-RsWRKY35 | WGD | 0.268067 | 2.10357 | 0.127434 |
| RsWRKY22-RsWRKY24 | WGD | 0.402691 | 2.09699 | 0.192033 |
| RsWRKY22-RsWRKY52 | WGD | 0.335212 | 1.82063 | 0.184119 |
| RsWRKY24-RsWRKY52 | WGD | 0.341843 | 1.90652 | 0.179302 |
| RsWRKY27-RsWRKY31 | WGD | 0.444906 | 1.74913 | 0.254359 |
| RsWRKY28-RsWRKY32 | WGD | 0.441321 | 3.15661 | 0.139809 |
| RsWRKY37-RsWRKY47 | WGD | 0.195994 | 0.743608 | 0.263572 |
| RsWRKY49-RsWRKY3 | Transposed | 0.180982 | 0.829485 | 0.218186 |
| RsWRKY43-RsWRKY4 | Transposed | 0.461986 | 3.62365 | 0.127492 |
| RsWRKY53-RsWRKY5 | Transposed | 0.226671 | 0.941693 | 0.240706 |
| RsWRKY21-RsWRKY9 | Transposed | 0.841286 | 1.92451 | 0.437143 |
| RsWRKY16-RsWRKY11 | Transposed | 0.636402 | 4.39191 | 0.144903 |
| RsWRKY41-RsWRKY18 | Transposed | 0.542645 | 0.998932 | 0.543226 |
| RsWRKY21-RsWRKY29 | Transposed | 0.607068 | 3.9559 | 0.153459 |
| RsWRKY51-RsWRKY29 | Transposed | 0.587673 | 4.13118 | 0.142253 |
| RsWRKY30-RsWRKY39 | Transposed | 0.265375 | 3.19294 | 0.083113 |
| RsWRKY57-RsWRKY54 | Transposed | 0.010566 | 0.0324457 | 0.325664 |
| RsWRKY1-RsWRKY25 | Dispersed | 0.65138 | 4.11454 | 0.158312 |
| RsWRKY4-RsWRKY48 | Dispersed | 0.325216 | 3.55886 | 0.091382 |
| RsWRKY45-RsWRKY4 | Dispersed | 0.381289 | N | 0.003851 |
| RsWRKY46-RsWRKY4 | Dispersed | 0.431817 | 3.56504 | 0.121125 |
| RsWRKY23-RsWRKY12 | Dispersed | 0.60194 | 1.92618 | 0.312505 |
| RsWRKY14-RsWRKY43 | Dispersed | 0.681244 | 3.65619 | 0.186326 |
| RsWRKY15-RsWRKY48 | Dispersed | 0.467646 | 1.70242 | 0.274695 |
| RsWRKY22-RsWRKY36 | Dispersed | 0.623376 | 4.26597 | 0.146127 |
| RsWRKY24-RsWRKY36 | Dispersed | 0.623717 | 1.72795 | 0.360957 |
| RsWRKY26-RsWRKY44 | Dispersed | 0.628186 | 4.3572 | 0.144172 |
| RsWRKY28-RsWRKY45 | Dispersed | 0.493253 | 3.51864 | 0.140183 |
| RsWRKY30-RsWRKY45 | Dispersed | 0.42428 | 3.23635 | 0.131098 |
| RsWRKY34-RsWRKY44 | Dispersed | 0.360469 | 1.65042 | 0.218411 |
| RsWRKY36-RsWRKY54 | Dispersed | 0.458786 | 1.50293 | 0.305262 |
| RsWRKY52-RsWRKY36 | Dispersed | 0.596921 | 4.3706 | 0.136576 |
| RsWRKY38-RsWRKY45 | Dispersed | 0.260053 | 1.70784 | 0.15227 |
| RsWRKY48-RsWRKY39 | Dispersed | 0.658747 | 3.82209 | 0.172352 |
| RsWRKY55-RsWRKY42 | Dispersed | 0.14514 | 0.195974 | 0.740606 |
| RsWRKY56-RsWRKY42 | Dispersed | 0.239831 | 0.650021 | 0.368959 |
| RsWRKY43-RsWRKY48 | Dispersed | 0.545615 | 3.93671 | 0.138597 |
| RsWRKY44-RsWRKY50 | Dispersed | 0.563524 | 4.36355 | 0.129143 |
| RsWRKY6-RsWRKY7 | Tandem | 0.25789 | 0.856657 | 0.301042 |
| RsWRKY18-RsWRKY19 | Tandem | 0.165611 | 0.289022 | 0.573003 |

Table S4. The orthologous relationships of the *WRKY* genes between *Arabidopsis* and azalea.

| Gene 1 | Gene 2 |
| --- | --- |
| *RsWRKY18* | AT2G40750 |
| *RsWRKY17* | AT2G38470 |
| *RsWRKY18* | AT3G56400 |
| *RsWRKY22* | AT1G62300 |
| *RsWRKY23* | AT2G04880 |
| *RsWRKY20* | AT2G23320 |
| *RsWRKY22* | AT4G22070 |
| *RsWRKY24* | AT4G22070 |
| *RsWRKY25* | AT4G26640 |
| *RsWRKY26* | AT4G26440 |
| *RsWRKY22* | AT4G04450 |
| *RsWRKY26* | AT5G56270 |
| *RsWRKY32* | AT1G64000 |
| *RsWRKY28* | AT1G64000 |
| *RsWRKY27* | AT2G46400 |
| *RsWRKY28* | AT2G46130 |
| *RsWRKY30* | AT2G44745 |
| *RsWRKY31* | AT2G46400 |
| *RsWRKY32* | AT2G46130 |
| *RsWRKY31* | AT4G11070 |
| *RsWRKY29* | AT4G01250 |
| *RsWRKY31* | AT4G23810 |
| *RsWRKY27* | AT4G23810 |
| *RsWRKY29* | AT4G23550 |
| *RsWRKY32* | AT5G41570 |
| *RsWRKY28* | AT5G41570 |
| *RsWRKY44* | AT1G13960 |
| *RsWRKY47* | AT1G80840 |
| *RsWRKY46* | AT1G69310 |
| *RsWRKY44* | AT2G03340 |
| *RsWRKY2* | AT2G30250 |
| *RsWRKY2* | AT2G38470 |
| *RsWRKY3* | AT2G30590 |
| *RsWRKY52* | AT1G62300 |
| *RsWRKY53* | AT1G29280 |
| *RsWRKY51* | AT1G30650 |
| *RsWRKY48* | AT2G47260 |
| *RsWRKY51* | AT2G34830 |
| *RsWRKY50* | AT2G30250 |
| *RsWRKY52* | AT4G04450 |
| *RsWRKY52* | AT4G22070 |
| *RsWRKY5* | AT1G29280 |
| *RsWRKY6* | AT2G25000 |
| *RsWRKY6* | AT4G31800 |
| *RsWRKY4* | AT5G49520 |
| *RsWRKY8* | AT2G40750 |
| *RsWRKY8* | AT3G56400 |
| *RsWRKY9* | AT5G52830 |
| *RsWRKY10* | AT5G24110 |
| *RsWRKY15* | AT2G47260 |
| *RsWRKY11* | AT4G26440 |
| *RsWRKY12* | AT4G26640 |
| *RsWRKY15* | AT5G49520 |
| *RsWRKY36* | AT1G18860 |
| *RsWRKY36* | AT1G69810 |
| *RsWRKY37* | AT1G80840 |
| *RsWRKY34* | AT1G13960 |
| *RsWRKY35* | AT2G23320 |
| *RsWRKY34* | AT2G03340 |
| *RsWRKY34* | AT3G01080 |
| *RsWRKY38* | AT3G01970 |
| *RsWRKY39* | AT4G39410 |
| *RsWRKY38* | AT5G13080 |

Table S5. The orthologous relationships of the *WRKY* genes between rice and azalea.

| Gene 1 | Gene 2 |
| --- | --- |
| *RsWRKY17* | LOC_Os01g61080 |
| *RsWRKY17* | LOC_Os05g39720 |
| *RsWRKY17* | LOC_Os05g27730 |
| *RsWRKY18* | LOC_Os05g25770 |
| *RsWRKY26* | LOC_Os08g38990 |
| *RsWRKY26* | LOC_Os09g30400 |
| *RsWRKY29* | LOC_Os01g43550 |
| *RsWRKY27* | LOC_Os01g46800 |
| *RsWRKY31* | LOC_Os01g46800 |
| *RsWRKY30* | LOC_Os04g46060 |
| *RsWRKY29* | LOC_Os05g50700 |
| *RsWRKY47* | LOC_Os02g08440 |
| *RsWRKY46* | LOC_Os03g55080 |
| *RsWRKY2* | LOC_Os01g61080 |
| *RsWRKY3* | LOC_Os12g40570 |
| *RsWRKY3* | LOC_Os03g53050 |
| *RsWRKY3* | LOC_Os03g58420 |
| *RsWRKY2* | LOC_Os05g39720 |
| *RsWRKY50* | LOC_Os01g61080 |
| *RsWRKY50* | LOC_Os05g27730 |
| *RsWRKY50* | LOC_Os05g39720 |
| *RsWRKY5* | LOC_Os01g54600 |
| *RsWRKY8* | LOC_Os03g21710 |
| *RsWRKY11* | LOC_Os08g38990 |

Table S6. The differentially expressed genes at bud stage.

| Gene Name | Average FPKM | | | Fold change (PF/WF) | Fold change (RF/WF) |
| --- | --- | --- | --- | --- | --- |
|  | WF_bud | PF_bud | RF_bud |  |  |
| RsWRKY21 | 1.200277098 | 6.460129716 | 3.427715228 | 5.382198601 | 2.85576992 |
| RsWRKY24 | 0.075410027 | 0.177191027 | 0.241592442 | 2.349701142 | 3.20371774 |
| RsWRKY33 | 0.244960459 | 0.636797297 | 0.82672056 | 2.599592192 | 3.37491431 |
| RsWRKY39 | 0.181188298 | 0.530765777 | 0.376568396 | 2.929360135 | 2.07832625 |
| RsWRKY2 | 45.51001339 | 12.58862677 | 11.34114283 | 0.27661224 | 0.24920104 |
| RsWRKY7 | 1.776934551 | 0.295616055 | 0.320985669 | 0.166362939 | 0.18064012 |
| RsWRKY17 | 22.68560962 | 6.613437093 | 3.810306097 | 0.29152565 | 0.16796137 |
| RsWRKY18 | 4.859619799 | 2.084714673 | 2.146489824 | 0.428987196 | 0.44169913 |
| RsWRKY26 | 5.486456771 | 1.701407133 | 0.76693388 | 0.310110369 | 0.13978674 |
| RsWRKY35 | 71.68634196 | 19.86592208 | 22.26547044 | 0.277122832 | 0.31059571 |
| RsWRKY36 | 2.308118188 | 0.157468556 | 0.434488999 | 0.068223784 | 0.18824383 |
| RsWRKY37 | 34.76542491 | 3.921503227 | 3.336765898 | 0.112798944 | 0.09597944 |
| RsWRKY38 | 1.466555103 | 0 | 0 | 0 | 0 |
| RsWRKY45 | 123.6951046 | 2.178348282 | 1.563080206 | 0.017610626 | 0.01263656 |
| RsWRKY47 | 157.2363963 | 37.51732105 | 30.91130875 | 0.238604559 | 0.19659131 |
| RsWRKY50 | 212.5856315 | 47.68537669 | 65.46214733 | 0.224311382 | 0.30793308 |
| RsWRKY52 | 26.47854784 | 3.620679875 | 2.539912564 | 0.136740122 | 0.09592341 |

Table S7. The differentially expressed genes at full bloom stage.

| Gene Name | Average FPKM | | | Fold change (PF/WF) | Fold change (RF/WF) |
| --- | --- | --- | --- | --- | --- |
|  | WF_full bloom | PF_full bloom | RF_full bloom |  |  |
| RsWRKY10 | 0.067848909 | 1.344422517 | 0.137813289 | 19.81494672 | 2.03117915 |
| RsWRKY27 | 2.203448591 | 7.254139661 | 4.884529397 | 3.292175589 | 2.216765763 |
| RsWRKY41 | 0.364794279 | 1.044639739 | 1.454576093 | 2.863640683 | 3.987387344 |
| RsWRKY51 | 0.017859885 | 0.132536891 | 0.089390728 | 7.420926496 | 5.005112276 |
| RsWRKY13 | 1.269240762 | 0.298465823 | 0.58646286 | 0.23515304 | 0.46205801 |
| RsWRKY25 | 1.206964806 | 0.546235896 | 0.558310462 | 0.452569863 | 0.462573937 |
| RsWRKY26 | 1.810738385 | 0.476570929 | 0.316294249 | 0.263191488 | 0.174676945 |
| RsWRKY29 | 2.503221644 | 0.949348371 | 0.64164926 | 0.379250624 | 0.256329383 |
| RsWRKY49 | 1.564543727 | 0.558745731 | 0.242059446 | 0.357130147 | 0.15471568 |

Table S8. The gene ontology (GO) analysis of candidate *RsWRKY* genes.

| Class | GO Term | GO_ID | P_value | Gene Ratio | Enrichment Score |
| --- | --- | --- | --- | --- | --- |
| Molecular function | transcription regulator activity | GO:0140110 | 1.72E-17 | 1 | 14.31758721 |
| Molecular function | sequence-specific DNA binding | GO:0043565 | 2.91E-17 | 1 | 25.42064516 |
| Molecular function | DNA-binding transcription factor activity | GO:0003700 | 4.62E-17 | 1 | 15.72306464 |
| Molecular function | nucleic acid binding | GO:0003676 | 7.38E-17 | 1 | 4.464309993 |
| Molecular function | DNA binding | GO:0003677 | 7.65E-17 | 1 | 8.830569251 |
| Molecular function | transcription regulatory region nucleic acid binding | GO:0001067 | 7.4E-16 | 0.52 | 25.87 |
| Molecular function | heterocyclic compound binding | GO:1901363 | 2.22E-10 | 1 | 2.53486876 |
| Molecular function | organic cyclic compound binding | GO:0097159 | 1.99E-10 | 1 | 2.532587736 |
| Molecular function | binding | GO:0005488 | 0.000121 | 1 | 1.46388765 |
| Cellular component | nucleus | GO:0005634 | 0.00000256 | 1 | 3.359493054 |
| Cellular component | intracellular membrane-bounded organelle | GO:0043231 | 0.03367797 | 1 | 1.536677815 |
| Cellular component | membrane-bounded organelle | GO:0043227 | 0.023824234 | 1 | 1.529685939 |
| Cellular component | intracellular organelle | GO:0043229 | 0.03299346 | 1 | 1.459242007 |
| Cellular component | organelle | GO:0043226 | 0.027168363 | 1 | 1.45600507 |
| Biological process | response to organonitrogen compound | GO:0010243 | 1.32E-17 | 0.64 | 32.61508982 |
| Biological process | regulation of cellular metabolic process | GO:0031323 | 1.59E-17 | 1 | 5.441496164 |
| Biological process | response to nitrogen compound | GO:1901698 | 1.92E-17 | 0.64 | 20.868659 |
| Biological process | heterocycle biosynthetic process | GO:0018130 | 2.42E-17 | 1 | 5.504851229 |
| Biological process | regulation of primary metabolic process | GO:0080090 | 2.61E-17 | 1 | 5.62677686 |
| Biological process | gene expression | GO:0010467 | 3.05E-17 | 1 | 4.276633166 |
| Biological process | regulation of RNA biosynthetic process | GO:2001141 | 4.38E-17 | 1 | 7.534749889 |
| Biological process | obsolete regulation of cellular macromolecule biosynthetic process | GO:2000112 | 8.41E-17 | 0.96 | 8.837295836 |
| Biological process | cellular nitrogen compound biosynthetic process | GO:0044271 | 1.66E-16 | 1 | 4.651817436 |
| Biological process | regulation of nucleobase-containing compound metabolic process | GO:0019219 | 1.79E-16 | 1 | 6.775875796 |
| Biological process | regulation of metabolic process | GO:0019222 | 2.06E-16 | 1 | 4.915102512 |
| Biological process | organic cyclic compound biosynthetic process | GO:1901362 | 2.18E-16 | 1 | 5.111411411 |
| Biological process | regulation of nucleic acid-templated transcription | GO:1903506 | 2.54-16 | 1 | 7.534749889 |
| Biological process | regulation of nitrogen compound metabolic process | GO:0051171 | 7.04E-16 | 1 | 5.752281176 |
| Biological process | aromatic compound biosynthetic process | GO:0019438 | 9.35E-16 | 1 | 5.424155513 |
| Biological process | RNA biosynthetic process | GO:0032774 | 2.17E-15 | 1 | 7.077338877 |
| Biological process | DNA-templated transcription | GO:0006351 | 2.17E-15 | 1 | 7.197040169 |
| Biological process | nucleobase-containing compound biosynthetic process | GO:0034654 | 2.17E-15 | 1 | 6.13148415 |
| Biological process | regulation of macromolecule metabolic process | GO:0060255 | 2.17E-15 | 1 | 5.372790404 |
| Biological process | macromolecule biosynthetic process | GO:0009059 | 2.17E-15 | 1 | 4.580462863 |
| Biological process | nucleic acid-templated transcription | GO:0097659 | 2.17E-15 | 1 | 7.118778754 |
| Biological process | regulation of biosynthetic process | GO:0009889 | 3.16E-15 | 1 | 6.489134579 |
| Biological process | RNA metabolic process | GO:0016070 | 3.16E-15 | 1 | 4.383466392 |
| Biological process | regulation of gene expression | GO:0010468 | 3.16E-15 | 1 | 6.280811808 |
| Biological process | regulation of macromolecule biosynthetic process | GO:0010556 | 3.16E-15 | 1 | 6.882733522 |
| Biological process | regulation of RNA metabolic process | GO:0051252 | 4.01E-15 | 1 | 7.142677298 |
| Biological process | regulation of cellular biosynthetic process | GO:0031326 | 4.01E-15 | 1 | 6.571814672 |
| Biological process | regulation of DNA-templated transcription | GO:0006355 | 4.01E-15 | 1 | 7.636159713 |
| Biological process | response to chitin | GO:0010200 | 4.01E-15 | 0.6 | 65.46538462 |
| Biological process | nucleic acid metabolic process | GO:0090304 | 3.13E-14 | 1 | 3.802725648 |
| Biological process | nucleobase-containing compound metabolic process | GO:0006139 | 2.68E-13 | 1 | 3.485053235 |
| Biological process | regulation of cellular process | GO:0050794 | 3.63E-13 | 1 | 3.439280663 |
| Biological process | cellular biosynthetic process | GO:0044249 | 3.57E-13 | 1 | 3.437197092 |
| Biological process | heterocycle metabolic process | GO:0046483 | 1.7E-12 | 1 | 3.226118271 |
| Biological process | organic substance biosynthetic process | GO:1901576 | 1.95E-12 | 1 | 3.204857842 |
| Biological process | cellular aromatic compound metabolic process | GO:0006725 | 2.72E-12 | 1 | 3.159057164 |
| Biological process | biosynthetic process | GO:0009058 | 3.47E-12 | 1 | 3.124839361 |
| Biological process | regulation of biological process | GO:0050789 | 5.21E-12 | 1 | 3.071273908 |
| Biological process | organic cyclic compound metabolic process | GO:1901360 | 6.56E-12 | 1 | 3.040007144 |
| Biological process | cellular nitrogen compound metabolic process | GO:0034641 | 9.75E-12 | 1 | 2.989286969 |
| Biological process | response to xenobiotic stimulus | GO:0009410 | 1.02E-11 | 0.6 | 9.643626062 |
| Biological process | biological regulation | GO:0065007 | 8.32E-11 | 1 | 2.738696702 |
| Biological process | negative regulation of DNA-templated transcription | GO:0045892 | 2.2E-09 | 0.36 | 19.83029126 |
| Biological process | negative regulation of RNA biosynthetic process | GO:1902679 | 3.36E-09 | 0.36 | 18.85403077 |
| Biological process | negative regulation of nucleic acid-templated transcription | GO:1903507 | 3.36E-09 | 0.36 | 18.85403077 |
| Biological process | response to oxygen-containing compound | GO:1901700 | 6.59E-09 | 0.64 | 5.419621891 |
| Biological process | negative regulation of RNA metabolic process | GO:0051253 | 7.55E-09 | 0.36 | 17.06841226 |
| Biological process | obsolete negative regulation of cellular macromolecule biosynthetic process | GO:2000113 | 1.14E-08 | 0.36 | 16.2534748 |
| Biological process | negative regulation of nucleobase-containing compound metabolic process | GO:0045934 | 1.83E-08 | 0.36 | 15.35729323 |
| Biological process | defense response to bacterium | GO:0042742 | 4.29E-08 | 0.36 | 13.89469388 |
| Biological process | negative regulation of macromolecule biosynthetic process | GO:0010558 | 5.32E-08 | 0.36 | 13.52662252 |
| Biological process | negative regulation of cellular biosynthetic process | GO:0031327 | 9.93E-08 | 0.36 | 12.53079755 |
| Biological process | negative regulation of biosynthetic process | GO:0009890 | 0.000000114 | 0.36 | 12.30433735 |
| Biological process | defense response to fungus | GO:0050832 | 0.000000174 | 0.32 | 15.04618785 |
| Biological process | response to external stimulus | GO:0009605 | 0.000000182 | 0.56 | 5.263257869 |
| Biological process | response to bacterium | GO:0009617 | 0.000000217 | 0.36 | 11.34733333 |
| Biological process | response to organic substance | GO:0010033 | 0.000000238 | 0.64 | 4.183348694 |
| Biological process | macromolecule metabolic process | GO:0043170 | 0.000000484 | 1 | 1.911183472 |
| Biological process | negative regulation of gene expression | GO:0010629 | 0.000000673 | 0.36 | 9.88316129 |
| Biological process | negative regulation of nitrogen compound metabolic process | GO:0051172 | 0.000000709 | 0.36 | 9.804096 |
| Biological process | response to fungus | GO:0009620 | 0.000000784 | 0.32 | 12.18505593 |
| Biological process | negative regulation of cellular metabolic process | GO:0031324 | 0.00000121 | 0.36 | 9.118392857 |
| Biological process | nitrogen compound metabolic process | GO:0006807 | 0.00000244 | 1 | 1.782490313 |
| Biological process | response to salicylic acid | GO:0009751 | 0.00000246 | 0.24 | 19.08897196 |
| Biological process | response to stress | GO:0006950 | 0.00000244 | 0.68 | 3.241187342 |
| Biological process | negative regulation of macromolecule metabolic process | GO:0010605 | 0.00000606 | 0.36 | 7.463532278 |
| Biological process | defense response | GO:0006952 | 0.0000109 | 0.4 | 5.859208262 |
| Biological process | negative regulation of metabolic process | GO:0009892 | 0.000011 | 0.36 | 6.869461883 |
| Biological process | leaf senescence | GO:0010150 | 0.0000141 | 0.2 | 20.75731707 |
| Biological process | response to chemical | GO:0042221 | 0.0000141 | 0.64 | 3.070304397 |
| Biological process | plant organ senescence | GO:0090693 | 0.0000164 | 0.2 | 20.02470588 |
| Biological process | cellular metabolic process | GO:0044237 | 0.0000165 | 1 | 1.638209817 |
| Biological process | regulation of defense response | GO:0031347 | 0.0000236 | 0.24 | 12.49247706 |
| Biological process | defense response to other organism | GO:0098542 | 0.0000236 | 0.36 | 6.201983806 |
| Biological process | response to acid chemical | GO:0001101 | 0.0000372 | 0.4 | 5.054491463 |
| Biological process | primary metabolic process | GO:0044238 | 0.0000421 | 1 | 1.574997687 |
| Biological process | response to antibiotic | GO:0046677 | 0.0000476 | 0.24 | 10.95184987 |
| Biological process | response to stimulus | GO:0050896 | 0.0000475 | 0.8 | 2.167244947 |
| Biological process | negative regulation of cellular process | GO:0048523 | 0.0000856 | 0.36 | 5.241710864 |
| Biological process | response to nutrient levels | GO:0031667 | 0.000125 | 0.2 | 12.74981273 |
| Biological process | obsolete multi-organism process | GO:0051704 | 0.000127 | 0.4 | 4.33932441 |
| Biological process | organic substance metabolic process | GO:0071704 | 0.000177 | 1 | 1.480730753 |
| Biological process | response to other organism | GO:0051707 | 0.000181 | 0.36 | 4.717136259 |
| Biological process | response to external biotic stimulus | GO:0043207 | 0.000184 | 0.36 | 4.702655411 |
| Biological process | response to biotic stimulus | GO:0009607 | 0.00019 | 0.36 | 4.677526718 |
| Biological process | response to extracellular stimulus | GO:0009991 | 0.000198 | 0.2 | 11.38528428 |
| Biological process | biological process involved in interspecies interaction between organisms | GO:0044419 | 0.000253 | 0.36 | 4.492346041 |
| Biological process | regulation of response to stress | GO:0080134 | 0.00027 | 0.24 | 7.781028571 |
| Biological process | response to organic cyclic compound | GO:0014070 | 0.000367 | 0.24 | 7.347194245 |
| Biological process | regulation of response to stimulus | GO:0048583 | 0.000438 | 0.32 | 4.798872247 |
| Biological process | secondary metabolic process | GO:0019748 | 0.000634 | 0.2 | 8.706393862 |
| Biological process | plant organ development | GO:0099402 | 0.000677 | 0.32 | 4.46452459 |
| Biological process | metabolic process | GO:0008152 | 0.000692 | 1 | 1.392083095 |
| Biological process | negative regulation of biological process | GO:0048519 | 0.000711 | 0.36 | 3.863530895 |
| Biological process | leaf development | GO:0048366 | 0.000775 | 0.2 | 8.242615012 |
| Biological process | cellular response to stress | GO:0033554 | 0.001072215 | 0.32 | 4.132564492 |
| Biological process | system development | GO:0048731 | 0.00155409 | 0.4 | 3.113123 |
| Biological process | positive regulation of cellular metabolic process | GO:0031325 | 0.002769706 | 0.24 | 4.817264151 |
| Biological process | phyllome development | GO:0048827 | 0.003060959 | 0.2 | 5.920347826 |
| Biological process | positive regulation of biological process | GO:0048518 | 0.003323776 | 0.32 | 3.425610063 |
| Biological process | positive regulation of metabolic process | GO:0009893 | 0.004197647 | 0.24 | 4.369026738 |
| Biological process | multicellular organism development | GO:0007275 | 0.009785578 | 0.4 | 2.384728546 |
| Biological process | developmental process | GO:0032502 | 0.014572483 | 0.44 | 2.111429377 |
| Biological process | positive regulation of cellular process | GO:0048522 | 0.015696033 | 0.24 | 3.255011952 |
| Biological process | cellular process | GO:0009987 | 0.01790509 | 1 | 1.204003678 |
| Biological process | shoot system development | GO:0048367 | 0.019679012 | 0.2 | 3.629211087 |
| Biological process | multicellular organismal process | GO:0032501 | 0.023427526 | 0.4 | 2.085267994 |
| Biological process | anatomical structure development | GO:0048856 | 0.027139883 | 0.4 | 2.032965064 |
| Biological process | cellular response to stimulus | GO:0051716 | 0.032378848 | 0.4 | 1.966608897 |

Table S9. The detailed information of the predicted three-dimensional (3D) proteins.

| **Gene Name** | **Gene ID** | **GMQE** | **QMEANDisCo global** | **Template Identity** | **Oligo-State** | **Template** | **Templete Description** |
| --- | --- | --- | --- | --- | --- | --- | --- |
| RsWRKY21 | Rhsim06G0156700 | 0.11 | 0.62 ± 0.11 | 46.58% | Monomer | 2ayd.1.A | WRKY transcription factor 1 |
| RsWRKY24 | Rhsim06G0220000 | 0.11 | 0.67 ± 0.11 | 50.00% | Monomer | 2ayd.1.A | WRKY transcription factor 1 |
| RsWRKY33 | Rhsim07G0225700 | 0.15 | 0.67 ± 0.11 | 54.05% | Monomer | 2ayd.1.A | WRKY transcription factor 1 |
| RsWRKY39 | Rhsim10G0147700 | 0.19 | 0.67 ± 0.11 | 50.67% | Monomer | 2ayd.1.A | WRKY transcription factor 1 |
| RsWRKY2 | Rhsim01G0241100 | 0.07 | 0.62 ± 0.11 | 78.87% | Monomer | 1wj2.1.A | Probable WRKY transcription factor 4 |
| RsWRKY7 | Rhsim02G0213800 | 0.21 | 0.76 ± 0.11 | 78.48% | Monomer | 7z0u.1.A | WRKY transcription factor 18 |
| RsWRKY17 | Rhsim05G0226500 | 0.13 | 0.63 ± 0.11 | 83.10% | Monomer | 1wj2.1.A | Probable WRKY transcription factor 4 |
| RsWRKY18 | Rhsim05G0231400 | 0.11 | 0.49 ± 0.11 | 60.00% | Monomer | 6ir8.1.A | OsWRKY45 |
| RsWRKY26 | Rhsim06G0235700 | 0.05 | 0.64 ± 0.11 | 83.10% | Monomer | 1wj2.1.A | Probable WRKY transcription factor 4 |
| RsWRKY35 | Rhsim08G0098300 | 0.13 | 0.68 ± 0.11 | 53.42% | Monomer | 2ayd.1.A | WRKY transcription factor 1 |
| RsWRKY36 | Rhsim08G0158800 | 0.09 | 0.68 ± 0.11 | 52.05% | Monomer | 2ayd.1.A | WRKY transcription factor 1 |
| RsWRKY37 | Rhsim08G0205900 | 0.17 | 0.73 ± 0.10 | 82.05% | Monomer | 7z0r.1.A | WRKY transcription factor 18 |
| RsWRKY38 | Rhsim10G0005100 | 0.27 | 0.69 ± 0.11 | 52.70% | Monomer | 2ayd.1.A | WRKY transcription factor 1 |
| RsWRKY45 | Rhsim12G0094500 | 0.27 | 0.70 ± 0.11 | 54.05% | Monomer | 2ayd.1.A | WRKY transcription factor 1 |
| RsWRKY47 | Rhsim12G0187400 | 0.17 | 0.70 ± 0.10 | 80.77% | Monomer | 7z0u.1.A | WRKY transcription factor 18 |
| RsWRKY50 | Rhsim13G0063200 | 0.07 | 0.63 ± 0.11 | 83.10% | Monomer | 1wj2.1.A | Probable WRKY transcription factor 4 |
| RsWRKY52 | Rhsim13G0151900 | 0.09 | 0.68 ± 0.11 | 52.00% | Monomer | 2ayd.1.A | WRKY transcription factor 1 |
| RsWRKY10 | Rhsim03G0198800 | 0.1 | 0.61 ± 0.11 | 43.94% | Monomer | 2ayd.1.A | WRKY transcription factor 1 |
| RsWRKY27 | Rhsim07G0005900 | 0.13 | 0.61 ± 0.11 | 39.44% | Monomer | 2ayd.1.A | WRKY transcription factor 1 |
| RsWRKY41 | Rhsim11G0064200 | 0.18 | 0.44 ± 0.11 | 40.00% | Monomer | 6ir8.1.A | OsWRKY45 |
| RsWRKY51 | Rhsim13G0125600 | 0.07 | 0.56 ± 0.10 | 41.89% | Monomer | 7z0u.1.A | WRKY transcription factor 18 |
| RsWRKY13 | Rhsim04G0052400 | 0.09 | 0.65 ± 0.11 | 49.33% | Monomer | 2ayd.1.A | WRKY transcription factor 1 |
| RsWRKY25 | Rhsim06G0226600 | 0.08 | 0.75 ± 0.11 | 66.22% | Monomer | 2ayd.1.A | WRKY transcription factor 1 |
| RsWRKY29 | Rhsim07G0010600 | 0.14 | 0.60 ± 0.11 | 58.67% | Monomer | 7p8k.1.A | Disease resistance protein RRS1 |
| RsWRKY49 | Rhsim13G0061600 | 0.14 | 0.66 ± 0.11 | 56.16% | Monomer | 2ayd.1.A | WRKY transcription factor 1 |
